# Machine learning approaches for risk prediction after percutaneous coronary intervention: a systematic review and meta-analysis

**DOI:** 10.1093/ehjdh/ztae074

**Published:** 2024-10-14

**Authors:** Ammar Zaka, Daud Mutahar, James Gorcilov, Aashray K Gupta, Joshua G Kovoor, Brandon Stretton, Naim Mridha, Gopal Sivagangabalan, Aravinda Thiagalingam, Clara K Chow, Sarah Zaman, Rohan Jayasinghe, Pramesh Kovoor, Stephen Bacchi

**Affiliations:** Department of Cardiology, Gold Coast University Hospital, 1 Hospital Boulevard, Southport, QLD 4215, Australia; Faculty of Health Sciences and Medicine, Bond University, 14 University Drive, Robina, QLD 4216, Australia; Faculty of Health Sciences and Medicine, Bond University, 14 University Drive, Robina, QLD 4216, Australia; University of Adelaide, Adelaide, SA 5005, Australia; Royal North Shore Hospital, Reserve Rd, St Leonards, NSW 2065, Australia; University of Adelaide, Adelaide, SA 5005, Australia; Ballarat Base Hospital, 1 Drummond St N, Ballarat Central, VIC 3350, Australia; University of Adelaide, Adelaide, SA 5005, Australia; Department of Cardiology, The Prince Charles Hospital, 627 Rode Rd, Chermside, QLD 4032, Australia; University of Notre Dame, 128-140 Broadway, Chippendale, NSW 2007, Australia; Department of Cardiology, Westmead Hospital, Cnr Hawkesbury Road and Darcy Rd, Westmead, NSW 2145, Australia; Department of Cardiology, Westmead Hospital, Cnr Hawkesbury Road and Darcy Rd, Westmead, NSW 2145, Australia; Faculty of Medicine and Health, Westmead Applied Research Centre, University of Sydney, NSW, Australia; Department of Cardiology, Westmead Hospital, Cnr Hawkesbury Road and Darcy Rd, Westmead, NSW 2145, Australia; Faculty of Medicine and Health, Westmead Applied Research Centre, University of Sydney, NSW, Australia; Department of Cardiology, Westmead Hospital, Cnr Hawkesbury Road and Darcy Rd, Westmead, NSW 2145, Australia; Faculty of Medicine and Health, Westmead Applied Research Centre, University of Sydney, NSW, Australia; Department of Cardiology, Gold Coast University Hospital, 1 Hospital Boulevard, Southport, QLD 4215, Australia; Department of Cardiology, Westmead Hospital, Cnr Hawkesbury Road and Darcy Rd, Westmead, NSW 2145, Australia; Faculty of Medicine and Health, Westmead Applied Research Centre, University of Sydney, NSW, Australia; Massachusetts General Hospital, 55 Fruit St, Boston, MA 02114, USA

**Keywords:** Machine learning, Artificial intelligence, Percutaneous coronary intervention, Coronary artery disease

## Abstract

**Aims:**

Accurate prediction of clinical outcomes following percutaneous coronary intervention (PCI) is essential for mitigating risk and peri-procedural planning. Traditional risk models have demonstrated a modest predictive value. Machine learning (ML) models offer an alternative risk stratification that may provide improved predictive accuracy.

**Methods and results:**

This study was reported according to the Preferred Reporting Items for Systematic Reviews and Meta-Analyses, Critical Appraisal and Data Extraction for Systematic Reviews of Prediction Modelling Studies and Transparent Reporting of a multivariable prediction model for Individual Prognosis or Diagnosis guidelines. PubMed, EMBASE, Web of Science, and Cochrane databases were searched until 1 November 2023 for studies comparing ML models with traditional statistical methods for event prediction after PCI. The primary outcome was comparative discrimination measured by *C*-statistics with 95% confidence intervals (CIs) between ML models and traditional methods in estimating the risk of all-cause mortality, major bleeding, and the composite outcome major adverse cardiovascular events (MACE). Thirty-four models were included across 13 observational studies (4 105 916 patients). For all-cause mortality, the pooled *C*-statistic for top-performing ML models was 0.89 (95%CI, 0.84–0.91), compared with 0.86 (95% CI, 0.80–0.93) for traditional methods (*P* = 0.54). For major bleeding, the pooled *C*-statistic for ML models was 0.80 (95% CI, 0.77–0.84), compared with 0.78 (95% CI, 0.77–0.79) for traditional methods (*P* = 0.02). For MACE, the *C*-statistic for ML models was 0.83 (95% CI, 0.75–0.91), compared with 0.71 (95% CI, 0.69–0.74) for traditional methods (*P* = 0.007). Out of all included models, only one model was externally validated. Calibration was inconsistently reported across all models. Prediction Model Risk of Bias Assessment Tool demonstrated a high risk of bias across all studies.

**Conclusion:**

Machine learning models marginally outperformed traditional risk scores in the discrimination of MACE and major bleeding following PCI. While integration of ML algorithms into electronic healthcare systems has been hypothesized to improve peri-procedural risk stratification, immediate implementation in the clinical setting remains uncertain. Further research is required to overcome methodological and validation limitations.

## Graphical Abstract

**Graphical Abstract:**
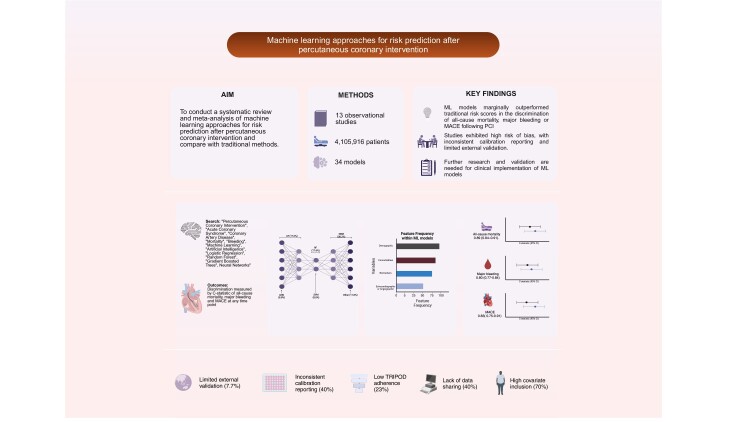

## Introduction

Percutaneous coronary intervention (PCI) is among the most frequently performed procedures globally.^1^ Procedural risk for patients is becoming increasingly pertinent due to growing indications and rates of concurrent comorbidities.^2,3^ Accurate prediction of clinical outcomes following PCI is crucial for identifying high-risk patients, robust peri-procedural planning, and facilitating informed consent.^4–6^

Traditional approaches for risk prediction including the Global Registry of Acute Coronary Events (GRACE) and Synergy Between PCI with TAXUS and Cardiac Surgery (SYNTAX) II risk scores have relied heavily on regression-based models and draw on routinely measured clinical variables including blood pressure, lipid levels, and smoking status or angiographic characteristics.^5–12^ These conventional risk scores are fixed, do not integrate the growing volume of patient data, and are contingent on statistical assumptions for valid application.^13,14^ These models have demonstrated a modest ability to determine the risk of major adverse cardiovascular events (MACE).^5–12^ Despite weak recommendations in contemporary guidelines, these scores are widely underutilized, with registry data demonstrating only 57% of patients undergoing PCI were evaluated using a contemporary risk score.^15–17^

Machine learning (ML), a subset of artificial intelligence, leverages algorithms to discern non-linear patterns in extensive data sets for outcome prediction.^18–20^ Contemporary ML models have demonstrated inconsistent results in the literature when compared with standard logistical regression.^21–24^ Machine learning algorithms encompass supervised, unsupervised, and reinforcement learning and are designed to evolve with the addition of new data, allowing for continuous refinement of predictive models.^25–27^ Supervised ML aligns predictor variables with a known outcome^27^ and includes decision trees, support vector machines (SVMs), and, by some definitions, standard epidemiological approaches such as logistic regression (LR).^27,28^ To ensure clinical applicability and mitigate the risk of overfitting, risk prediction tools developed through ML undergo rigorous validation, employing metrics such as discrimination, calibration, and net reclassification improvement to assess their efficacy against traditional models.^29^

Previous meta-analyses have evaluated the predictive value of ML algorithms in cardiovascular medicine.^21,30–34^ Currently, there is no meta-analysis that synthesizes the evidence comparing performance of ML-based models and traditional methods in patients undergoing PCI. The aim of this study was to conduct a systematic review and meta-analysis comparing the performance of ML algorithms with traditional risk scores for predicting clinical outcomes after PCI.

## Methods

This systematic review and meta-analysis was reported according to the Preferred Reporting Items for Systematic Reviews and Meta-Analyses, Critical Appraisal and Data Extraction for Systematic Reviews of Prediction Modelling Studies, and Transparent Reporting of a multivariable prediction model for Individual Prognosis or Diagnosis (TRIPOD) guidelines.^35–38^ This review was registered with PROSPERO (CRD42024499144) and did not require institutional board review.

### Search strategy

A comprehensive search strategy was designed and conducted using PubMed, Embase, Web of Science, and Cochrane databases. No time limit to start date was applied, and the search was conducted up to 1 November 2023. We manually searched the references cited in the previous reviews and other important studies related to this subject. We did not need to contact the corresponding authors of the studies, as the relevant information was easily accessible from the original studies. The search strategy, search terms used, and inclusion and exclusion criteria are detailed in the Supplementary material.

### Study eligibility

We included all studies that predicted all-cause mortality, bleeding, and the composite outcome MACE (as defined by original studies) following an index admission for PCI using an ML algorithm and traditional risk scores (as defined by the original studies). Studies were excluded if ML and traditional risk score models were not both evaluated on the same data set. We also excluded review articles, abstracts without full text available, and studies not in English. No minimum follow-up time was specified.

### Outcomes

The primary outcome assessed in this study was the comparative discrimination between ML models and traditional risk scores for prediction of all-cause mortality, major bleeding, and the composite outcome MACE at any time point. These clinical outcomes were defined according to the criteria used in the original studies. The *C*-statistic [area under the receiver operating characteristic (ROC) curve] was chosen as the preferred measure of discrimination as it provides a measure of model accuracy without specifying a diagnostic threshold.^39^ The *C*-statistic provides a probabilistic measure of the model’s discriminatory power, representing the likelihood that a randomly chosen case (e.g. an individual who develops an adverse event) has a higher predicted risk score than a randomly chosen non-case (e.g. an individual who does not develop the event). A *C*-statistic of 0.75 implies that a randomly chosen case has a higher risk score than a non-case 75% of the time. A score below 0.70 may indicate poor discrimination, while scores between 0.70–0.80 and 0.80–0.90 are deemed acceptable and excellent, respectively.^39^ Other measures of model performance including accuracy, sensitivity and specificity, calibration, risk reclassification analysis, likelihood ratio testing, and Bayes information criterion were also considered and however not formally assessed due to inconsistent reporting. These outcomes were consistent with those recommended previously.^40^

### Machine learning models

Based on the extracted data, ML algorithms were classified into six subgroups: LR-based algorithms, gradient boosted trees, random forest, SVMs, neural networks, and other mixed ML approaches (e.g. generalized additive models and Poisson regressions).^18,21^ Decision trees classify data by splitting it at nodes based on the best predictors, creating branches that lead to terminal leaf nodes. Gradient boosting machines combine multiple weak learners, typically tree-based, to build a strong predictor and minimize misclassification. Random forests aggregate numerous decision trees, with each tree voting for a category, and the forest prediction based on the majority vote. Support vector machines create a decision boundary, or hyperplane, between classes to enable classification, maximizing the margin between the closest data points. Artificial neural networks (ANNs) learn from labelled examples during training, using neurons arranged in input, hidden, and output layers to process and compute outputs based on the relative importance of inputs. Machine learning models were categorized based on the influence of covariates: low (traditional cardiovascular disease risk factors from detailed history), moderate (including non-traditional lab values), and high (requiring biobank or imaging data). This classification was consistent with a previous review by Liu *et al.*^21^

### Data extraction and quality assessment

Two reviewers (A.Z. and D.M.) screened all the titles and abstracts independently. This was performed with a free-to-use web application (Rayyan, Qatar Computing Research Institute, Ar-Rayyan, Qatar).^41^ Conflicts were resolved by inclusion of a third reviewer (A.K.G.). This process was followed by the full-text review of the selected articles by the two independent reviewers (A.Z. and D.M.). We then extracted the data from selected studies using a standardized, pilot-tested extraction template. Data were extracted by two independent reviewers (A.Z. and J.G.), and conflicts were resolved through discussion or a third reviewer (D.M.). The extracted data included data set characteristics, features of ML and traditional models, and performance metrics including *C*-statistics, sensitivity, specificity, predictive values, and calibration metrics. Studies were assessed based on their training, internal validation, and external validation methods. We did not need to contact authors for missing data. Two reviewers (D.M. and A.Z.) assessed quality of included models by using the Prediction Model Risk of Bias Assessment Tool (PROBAST).^42^ Disagreements between reviewers for classifications were resolved by consensus.

### Statistical analysis

All meta-analyses were conducted using a random-effects model to account for population, predictor variable, and outcome heterogeneity. For inclusion in the meta-analysis, studies needed to provide or allow the estimation of *C*-statistics with 95% confidence intervals (CIs) for both ML models and traditional risk scores. Pooled *C*-statistics from ML and traditional risk score models were compared and assessed for statistical significance, set at *P* < 0.05.^43^ In cases where studies evaluated several ML models or traditional risk scores derived from the same data set, the top-performing model (based on *C*-statistic or area under receiver operating characteristic [AUC-ROC])) was selected for inclusion in the meta-analysis to prevent unit-of-analysis errors. Based on previous methods, heterogeneity was not formally assessed and assumed to be present given variability in sample size, outcome time points, baseline demographics, and predictor variables.^44^ Given the inherent heterogeneity, small sample size, and potential for Type 1 error, prediction intervals were calculated using the Hartung–Knapp–Sidik–Jonkman method.^45^ Calibration analysis was not formally analysed due to diverse reporting metrics, including observed to expected (O/E) ratios, Hosmer–Lemeshow tests, and calibration plots. Brier scores were extracted but not used in calibration comparisons due to inconsistent interpretations in existing literature.^46,47^ Machine learning algorithms were classified into six subgroups, namely LR-based algorithms, random forest, gradient boosted trees, SVMs, neural networks, and other mixed ML approaches (e.g. generalized additive models and Poisson regressions).^18,21^ All calculations were performed using Review Manager 5.4 Cochrane Collaboration and R.

## Results

### Search results

The literature search yielded 5916 unique studies after removal of duplicates, and 13 studies were included in this systematic review and meta-analysis (see *Figure 2*).^2,48–59^ Detailed rationale for exclusion of full-text studies is provided in the Supplementary material.

**Figure 1:**
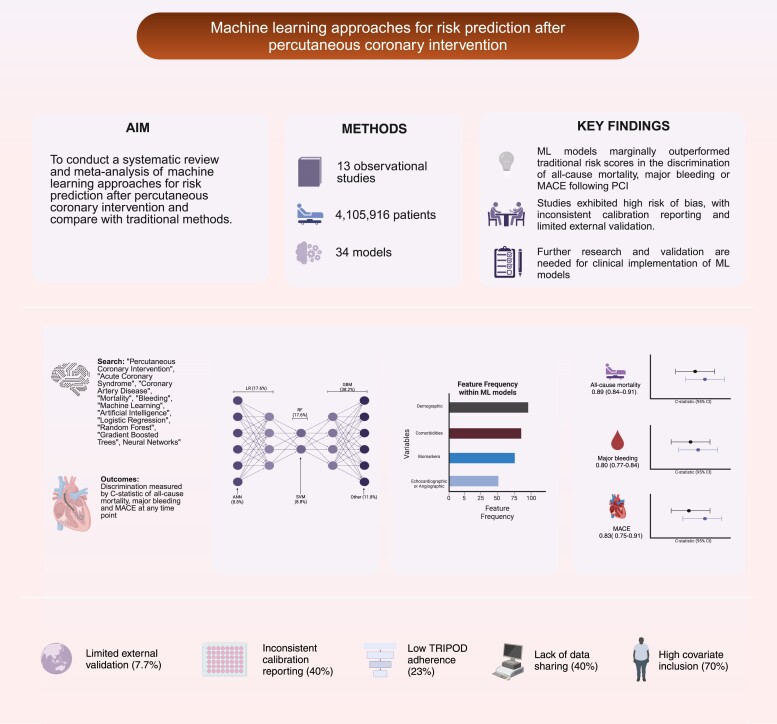
Central graphical abstract summary.

**Figure 2:**
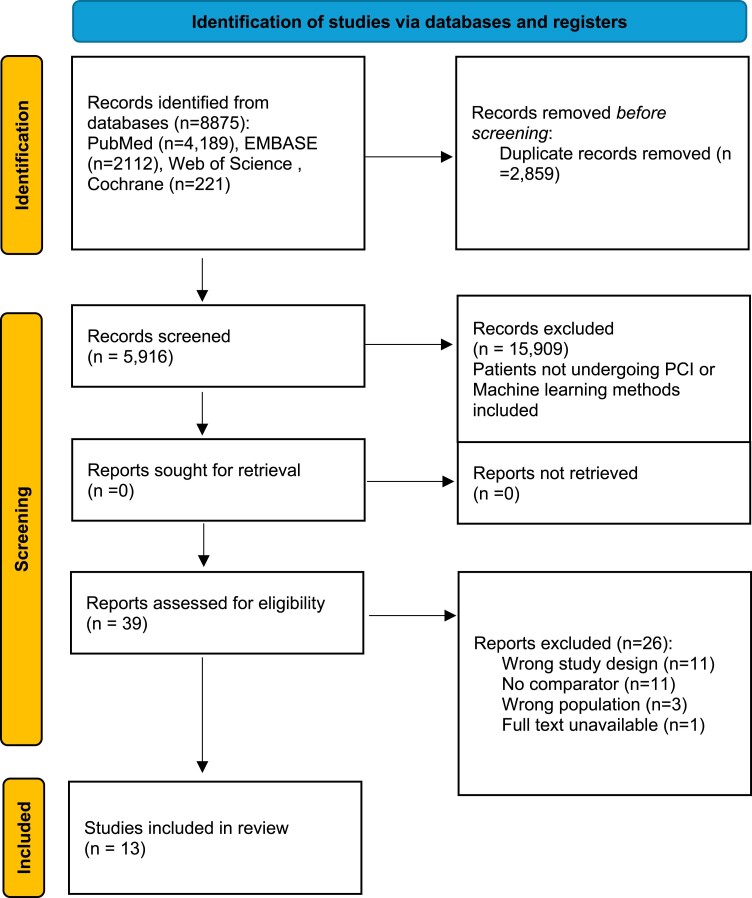
Study assessment and inclusion flowchart.

### Included studies

A total of 13 studies published between 2001 and 2023 were included in this systematic review (see *Table 1*). Three studies were prospective cohort studies, and the remaining were of retrospective cohort design. Cohort sizes ranged between 194 and 3 316 465 patients. One study reported externally validated performance measures, and the remaining studies undertook internal validation. Three studies evaluated models on the Mayo Clinic PCI Registry (*Table 1*). The most commonly reported traditional risk scores were the National Cardiovascular Data Registry (NCDR)-Cath PCI-based models (*n* = 3) and GRACE score (*n* = 2). Characteristics of included studies are further detailed in *Table 1*.

**Table 1:** Characteristics of included studies

Study	Registry	Design	Sample size	Outcome	Average follow-up	Age	Male	STEMI	NSTEACS	Stable angina	All-cause mortality	Bleeding	MACE
Călburean *et al.*^48^	Local PCI Registry of the Emergency Institute for Cardiovascular Diseases and Transplantation of Targu Mures	Prospective cohort study	2242	3-year all-cause mortality	3 years	64 years (median, IQR: 57–71)	1585 (70.6%)	569 (25.3%)	615 (27.4%)	1052 (46.9%)	336/2242 (14.9%)	NR	NR
Chao *et al.*^58^	Mayo Clinic Institutional NCDR-Cath PCI Registry January 2006–December 2018	Retrospective cohort study	17 356	Ischaemic stroke at 6 months post-PCI	NR	66.9 ± 12.5	11 104 (70.7%)	3090 (19.7%)	NR	NR	NR	NR	109/17 356 (0.6%) at 6 months
													132/17 356 (0.8%) at 1 year
													175/17 356 (1.0%) at 2 years
													264/17 356 (1.5%) at 5 years
Doll *et al.*^2^	Veterans Affairs Clinical Assessment Reporting and Tracking programme	Retrospective cohort study	70 471	30-day mortality post-PCI procedure		66.5 years (mean, SD: 9.1)	69 (98.1%)	4158 (5.9%)	15 141 (21.5%)	23 117 (32.8%)	1198/70 471 (1.7%)	NR	NR
Li *et al.*^49^	Department of Ultrasound Imaging and Cardiology, Renmin Hospital of Wuhan University, Wuhan, China in June 2012–June 2022	Single-centre, prospective analysis of retrospectively acquired myocardial contrast echocardiography studies	194	MACEs defined as cardiac death, hospitalization for congestive heart failure, re-infarction, stroke, and recurrent angina	6 months (medium: 333 days, Q1−Q3: 207–432 days)		34 (75.56%) for MACE group, 124 (83.22%) for event-free group	194 (100%)	0	0	5/194 (2.58%)	NR	45/194 (23.2%)
Mortazavi *et al.*^50^	NCDR-Cath PCI Registry (1 July 2009–1 April 2015)	Retrospective cohort study	3 316 465	Post-PCI major bleeding: in-hospital major bleeding within 72 h after PCI	NR	Median age of 65 years (IQR: 56–73	2 261 330 (68.1%)	554 240 (16.7%)	NR	NR	NR	149724/3 316 465 (4.5%)	NR
Niimi *et al.*^51^	Japan Cardiovascular Database-Keio Interhospital Cardiovascular Studies: a large, multicentre (*n* = 15) PCI registry in collaboration with NCDR-Cath PCI (July 2008–September 2020)	Retrospective cohort study	22 958	Peri-PCI adverse events	NR	70 years	18 213 (79.3%)	5083 (22.1%)	5163 (22.5%)	12 712 (55.4%)	529/22 958 (2.3%)	1784/22 958 (7.8%)	NR
				acute kidney injury									
				bleeding									
				In-hospital mortality									
Rayfield *et al.*^59^	Mayo Clinic Institutional NCDR-Cath PCI Registry: patients who underwent PCI between January 2003 and June 2018	Retrospective cohort study	15 603	Post-PCI bleeding (bleeding within 72 h of PCI and prior to hospital discharge; bleeding was defined according to NCDR	NR	67 ± 12.7	2922 (68.5%)	NR	NR	NR	NR	280/15 603 (1.8%)	NR
Resnic *et al.*^52^	PCI at the Division of Cardiovascular Medicine and Decision Systems Group, January 1997–31 December 1999	Prospective cohort study	4264	In-hospital mortality from any cause after coronary intervention	NR	64.3 ± 12.1	2922 (68.5%)	394 (9.2%)	NR	NR	91/4264 (2.1%)	NR	273/4264 (6.4%)
				Combined endpoints of in-hospital death, myocardial infarction, and same stay bypass surgery (MACE)									
Sampedro *et al.*^53^	Grupo de Analisis de la Cardiopatia Isquemica Aguda−3 RCTs; 263 STEMI patients enrolled from 20 Spanish hospitals	Retrospective cohort study	263	Stent restenosis of infarct-related lesion, defined as >50% narrowing of lumen diameter, at 12 months post-PCI in STEMI patients	12 months	NR	263 (100%)	263 (100%)	0	0	NR	NR	23/263 (9%)
Shi *et al.*^54^	Fuwai PCI Cohort (January to December 2013): Acute coronary syndrome (ACS) patients who underwent PCI at Fuwai Hospital (National Centre for Cardiovascular Diseases, Beijing, China) with a 1-year follow-up	Retrospective cohort study	6412	1-year all-cause mortality recurrent AMI Major bleeding (BARC Type 3 or 5 bleeding)	881 days (median; inter-quartile range: 807–944 days).	59 years (median; inter-quartile range: 51–66 years)	4925 (76.8%)	1439 (22.4%)	4973 (77.6%)		47/6412 (0.7%)	16/6412 (0.2%)	NR
									

Xiao *et al.*^55^	Enrolled patients diagnosed with AMI in the hospital information system of Zhuzhou Central Hospital PCI August 2018–December 2019	Retrospective cohort study	408	MACE: myocardial infarction, heart failure, kidney failure, coronary events, cerebrovascular events, and death	1.42 years	62.95 ± 12.98	317 (77.7%)	258 (63.2%)	NR	NR	NR	NR	125/408 (30.6%)
Zack *et al.*^56^	Mayo Clinic PCI Registry: patients undergoing PCI between January 2004 and December 2013	Retrospective cohort study	11 709	All-cause in-hospital mortality (death in hospital or within 3 days of discharge, 180 days), 30 days CHF readmission	NR	66.9 years	10 255 (71.5%)	NR	NR	NR	239/11 709 (2.0%)	NR	NR
Zhao *et al.*^57^	Multicentre China Acute Myocardial Infarction Registry (January 2013–30 June 2016)	Prospective cohort study	16 736	In-hospital major bleeding (BARC Type 3 or 5 of non-CABG-related bleeding)	NR	60.08 years	13 311 (79.5%)	NR	NR	NR	NR	70/16 736 (0.42%)	NR

AMI, acute myocardial infarction; CABG, coronary artery bypass grafting; IQR, inter-quartile range; LR, logistic regression; GAM, generalized additive model; SVM, support vector machine; RF, random forest; GB, gradient boosted tree; NN, neural network; NCDR, National Cardiovascular Data Registry; MACE, major adverse cardiac event; STEMI, ST-elevation myocardial infarction; RCT, randomized controlled trial.

### Model characteristics

The number of different ML models assessed within each study ranged from 1 to 6. In total, 34 models were assessed across the13 studies. Machine learning models assessed included gradient boosted trees (*n* = 13, 38.2%), random forests (*n* = 6, 17.6%), neural networks (*n* = 3, 8.8%), LR-based models (*n* = 5, 14.7%), SVMs (*n* = 3, 8.8%), and alternative or mixed statistical models (e.g. generalized additive models and proportional hazard models) (*n* = 4, 11.8%). Of the outcomes assessed, six studies assessed all-cause mortality, five studies assessed major bleeding events, four studies assessed the composite outcome MACE, and one study each assessed recurrent myocardial infarction, congestive heart failure, ischaemic stroke, and in-stent stenosis, precluding reliable meta-analysis. As given in *Table 2*, data transformation, feature scaling, or pre-processing was not described in four studies (30.8%). The method and threshold for low variance feature removal was described in two studies (15.4%). Other data pre-processing steps, such as over-sampling to address class imbalance, were utilized in only two studies (15.4%), while exploratory data analysis was conducted in just three studies (23.1%). The handling of missing data across the studies varied. Most models (61.5%) used multiple imputation techniques, with specific methods such as predictive mean matching (30.8%), chained equations (23.1%), and *k*-nearest neighbours (7.7%) for continuous variables and mode imputation for categorical variables (61.5%). Sensitivity analyses (38.5%) and robustness checks (38.5%) were commonly conducted to assess the impact of missing data. Some studies (30.8%) excluded patients with extensive missing data or specific variables with high missing rates to maintain data integrity. The number of included variables ranged from 6 to 410, including 178 distinct variables across all 13 studies. The most commonly reported baseline predictor variables from each model were extracted from all studies and are summarized in *Figure 2*. Age, gender, and diabetes mellitus emerged as the predominant variables, featuring in 100% of all models. Among biomarkers and laboratory values, serum creatinine was the most predominant variable (76.9%). Among echocardiographic variables, left ventricular ejection fraction (pre- and post-procedure) emerged as the predominant variable (69.2%). Among angiographic characteristics, lesion complexity (based on Society for Cardiovascular Angiography & Interventions lesion classification, SYNTAX score, and American Heart Association/American College of Cardiology [AHA/ACC] lesion classification) and degree of stenosis at time of PCI (as measured by quantitative coronary angiograph) emerged as predominant variables (61.5%, respectively). A subset of models (20.56%) incorporated traditional risk scores, such as NCDR-Cath PCI-based models (*n* = 3) and GRACE score (*n* = 2), into the ML model feature set. A detailed summary of baseline variables used in the ML models is provided in *Table 3*.

**Table 2:** Model characteristics

Study	Models	Traditional risk score	Variables	Handling of missing data	Split ratio of training to testing sample	Model evaluation	Data pre-processing techniques	*C*-statistic (AUC-ROC)	Calibration	Validation	ML software
Călburean *et al*.^48^	Gradient boosting with categorical features support (CatBoost)	GRACE score ACEF score SYNTAX II 2020 score Thrombolysis in Myocardial Infarction (TIMI) score	41 variables in the XGBoost model predicting MACEs	Used mean imputation for continuous variables Mode imputation for categorical variables Excluded cases with missing data for key variables Employed data augmentation techniques for imputation	70% training/30% testing	ROC AUC: provided as *C*-statistic PR AUC: also reported alongside ROC AUC Other metrics: F1 max score, MCC, NRI	Missing data imputation, exploratory data analysis: not explicitly mentioned, but pre-processing included translating raw data into structured formats for analysis	0.854 (0.835–0.875, *P<0.001*)	Brier score = 0.086	Single random split (70% training and 30% testing)	NR
Chao *et al.*^58^	RF	LR	45 variables	Used median imputation for continuous variables Mode imputation for categorical variables Excluded patients with extensive missing data Conducted sensitivity analysis on the impact of missing data	70% training and 30% testing	ROC AUC: provided as *C*-statistic Other metrics: calibration plots were used to assess model performance	Data transformation or feature scaling: normalization was carried out Handling low variance: features with missing values >20% were discarded, and discrete variables whose variance did not meet a set threshold were removed Missing data imputation: missing values were imputed for categorical and continuous variables	Ischaemic stroke at 6 months post-PCI: 0.87 (0.82–0.91, *P* = 0.001) Ischaemic stroke at 1 year post-PCI: 0.81 (0.76–0.86, *P* = 0.011) Ischaemic stroke at 2 years post-PCI: 0.77 (0.73–0.80, *P* = 0.01) Ischaemic stroke at 5 years post-PCI: 0.77 (0.74–0.79, *P* < 0.001)	No calibration reported	Single random split (70% training and 30% testing)	Python using the Scikit-learn library for ML
Doll *et al.*^2^	Coronary Artery Revascularization Therapy for Percutaneous Coronary Intervention (CART PCI) mortality model	NCDR-Cath PCI mortality model	14 variables in the final model after variable reduction and selection	Applied multiple imputation techniques Used predictive mean matching. Imputed missing values for each data set separately before merging. Ensured consistency by using the same imputation model across data sets	80% training/20% testing	ROC AUC: provided as *C*-statistic PR AUC: sensitivity, specificity, precision, NPV	Missing data imputation	0.93 (0.92–0.94, *P* < 0.001)	Calibration plot of Veteran Affairs (VA) PCI 30-day mortality model assessed on the development cohort (true rate of death % vs. predicted death %): slope = 1.025, indicating good performance at each decile of estimated risk	External validation (chronological split)	R, Empower Stats
Li *et al.*^49^	Microvascular blood flow + global longitudinal strain model Deep neural network	Visual microvascular perfusion method	23 variables	Handled missing data using multiple imputation Applied five iterations for imputation Used chained equations method for imputation Ensured robustness by checking the consistency of imputed data	75% training and 25% testing.	Sensitivity, specificity, accuracy, PPV, NPV for various threshold settings ROC AUC: provided as *C*-statistic PR AUC reported alongside ROC AUC	Data transformation or feature scaling: normalization was performed Missing data imputation: imputation for missing values was performed	0.90 (0.86–0.93)	Calibration was performed by visual calibration plot comparing predicted and actual probability of MACE, showing the predicted risks of MACE by the model agreed well with the actual event rates	Cross-validation (10-fold cross-validation)	Python with Scikit-learn for model development and TensorFlow for deep learning
Mortazavi *et al.*^50^	Blended model with gradient descent boosting XGBoost	NCDR bleeding risk model	59 variables	Used mean imputation for continuous variables Mode imputation for categorical variables Conducted sensitivity analysis to assess the impact of missing data Excluded patients with extensive missing data	80% training and 20% testing	ROC AUC: provided as *C*-statistic Sensitivity/specificity Other metrics: accuracy, calibration curves	Exploratory data analysis: not explicitly mentioned, but various pre-processing methods for preparing data as dichotomous variables were investigated	0.82 (0.82–0.82)	The blended model demonstrated a closer calibration than the existing full model (Brier score of 0.039 vs. 0.041) on a decile-based calibration plot (calculated from the five-fold cross-validation) and continuous calibration plot	Cross-validation (five-fold cross-validation)	R (version 4.0.4) with the tidymodels bundle of packages, xgboost (version 1.3.2.1), Partial Receiver Operating Characteristic Package in R (pROC) (version 1.17.0.1), verification (version 1.42), predictABEL (version 1.2.4), and mice (version 3.14.0)
Niimi *et al.*^51^	LR Extreme gradient boosting (XGBoost) model	NCDR-Cath PCI risk scores	15 variables	Imputed pre-procedural haemoglobin with post-procedural values Imputed pre-procedural creatinine with post-procedural values Used median imputation for continuous variables Mode imputation for categorical variables	70% training and 30% testing	ROC AUC: provided as *C*-statistic Other metrics: calibration plots	NR	LR Bleeding: *C*-statistic 0.75 (0.73–0.78, *P* = 0.64); AUC 0.289 In-hospital mortality: *C*-statistic 0.95 (0.94–0.97, *P* = 0.46); AUC 0.378 XGBoost Bleeding: *C*-statistic 0.79 (0.76–0.81, *P* < 0.001); AUC 0.393 In-hospital mortality: *C*-statistic 0.96 (0.94–0.97, *P* = 0.80); AUC 0.400	LR Brier scores Acute Kidney Injury (AKI): 0.064, bleeding: 0.084, in-hospital mortality: 0.019 XGBoost Brier scores AKI: 0.06, bleeding: 0.081, in-hospital mortality: 0.019 NCDR-Cath PCI risk scores Brier scores AKI: 0.064, bleeding: 0.087, in-hospital mortality: 0.021	Cross-validation (five-fold cross-validation)	R (version 4.0.4; R Project for Statistical Computing, Vienna, Austria) tidymodels (version 0.1.2) bundle of packages for data pre-processing, hyper-parameter tuning, learning, and performance metrics xgboost (version 1.3.2.1) for extreme gradient boosting pROC (version 1.17.0.1) for calculating *C*-statistics verification (version 1.42) for calculating Brier scores predictABEL (version 1.2.4) for calculating NRI mice (version 3.14.0) for multiple imputation
Rayfield *et al.*^59^	Artificial intelligence bleeding risk model Boosted classification tree algorithm	American College of Cardiology-Cath PCI bleeding risk model	108 variables	Used multiple imputation for missing data Applied predictive mean matching Ensured consistency in imputation across data sets Conducted robustness checks on imputed data sets	80% training and 20% testing	ROC AUC: provided as *C*-statistic Sensitivity/specificity PPV/NPV: mentioned as part of the model evaluation	NR	0.873 (*P* = 0.0243)	No calibration.	Cross-validation (five-fold cross-validation)	R (version 3.5.1) with the caret package (version 6.0–80) for augmented intelligence algorithms and data processing
Resnic *et al.*^52^	LR, NN	Simplified prognostic risk scoring systems	20 hidden nodes	Used forward and backward stepwise selection for model construction Excluded variables with high rates of missing data Conducted robustness checks on models without missing data adjustments Focused on maintaining a complete data set for key analysis variables	67% training and 33% testing	ROC AUC: provided as *C*-statistic	NR	LR In-hospital mortality: AUC 0.85 In-hospital death, myocardial infarction, or same-day bypass surgery (MACE): AUC 0.78 NN In-hospital mortality: AUC 0.83 In-hospital death, myocardial infarction, or same-day bypass surgery (MACE): AUC 0.81	LR Hosmer–Lemeshow In-hospital mortality: 13.060 NN Hosmer–Lemeshow In-hospital mortality: 7.170 Simplified prognostic risk scoring systems Hosmer–Lemeshow In-hospital mortality: 8.86	Single random split (random split into training and test sets)	STATA 6.0 for multiple LR models and risk score models Goodman’s NetProv4 software for feedforward ANNs
Sampedro *et al.*^53^	RF, extreme randomized trees, GB, SVM classifier, L2-regularized LR, non-regularized LR (LR_NOREG)	PRospective Evaluation of non-ST-elevation myocardial infarction Treatment Strategy On outcomes (PRESTO)-1 risk score PRESTO-2 risk score EVENT risk score	68 variables	Implemented multiple imputation techniques Applied imputation by chained equations Conducted separate imputations for each outcome Ensured consistency across imputed data sets	NR	ROC AUC: provided as *C*-statistic PR AUC: For ML model and clinical scores. Other metrics: sensitivity, specificity, precision, NPV at different thresholds	Data transformation or feature scaling: one-hot encoding for multi-category features Missing data imputation: median and mode imputation for missing values Over-sampling: use of *k*-fold cross-validation to handle imbalanced outputs	0.77 (0.66–0.89)	No calibration	Cross-validation (10-fold cross-validation with 20 repetitions)	Python with the scikit-learn library for implementing *k*-fold splitting, feature selection, and ML classifiers
Shi *et al.*^54^	PRAISE score Adaptive Boosting (AdaBoost)	GRACE Predicting Bleeding Complications in Patients Undergoing Dual Antiplatelet Therapy (PRECISE-DAPT) PARIS	98 variables	Employed multiple imputation using chained equations Conducted sensitivity analysis on imputed data sets Used mean imputation for continuous variables Mode imputation for categorical variables	NR	ROC AUC: provided as *C*-statistic Specificity, calibration	Data transformation or feature scaling: normalization Missing data imputation	1-year all-cause mortality: *C*-statistic 0.75 (0.67–0.83); AUC-ROC 0.75 Recurrent AMI: *C*-statistic 0.61 (0.52–0.69); AUC-ROC 0.61 Major bleeding (BARC Type 3 or 5 bleeding): *C*-statistic 0.62 (0.46–0.77); AUC-ROC 0.62	Calibration curves of the PRAISE score for all-cause mortality, recurrent acute myocardial infarction, and major bleeding were all under the perfect calibration line O:E ratios of all-cause mortality, MI, and major bleeding were 0.427, 0.260, and 0.106, respectively The Hosmer–Lemeshow goodness-of-fit test *P* values for all-cause mortality, recurrent AMI, and major bleeding were all <0.001	The study used X-tile method to split the cohort, but the validation process aligns with cross-validation principles	R (version 4.1.1, R Foundation for Statistical Computing, Vienna, Austria)
Xiao *et al.*^55^	DT, Naïve Bayes, SVM, RF, GB, MLP	LR	24 variables	Handled missing data using multiple imputation Applied *k*-nearest neighbours imputation for continuous variables Mode imputation for categorical variables Validated models on both original and imputed data sets	60% training and 40% testing	ROC AUC: provided as *C*-statistic Accuracy F1 score Other metrics: calibration plots	Data transformation or feature scaling: min–max scaling for normalization Handling low variance: removal of features with variance below 0.09 Missing data imputation: imputation for categorical and continuous variables Over-sampling: addressed class imbalance by providing weights proportional to class frequencies	0.664 (0.488–0.840) 0.733 (0.650–0.718) 0.717 (0.687–0.746) 0.749 (0.644–0.853) 0.737 (0.637–0.838) 0.663 (0.532–0.794)	Calibrated using Platt’s scaling, and the calibration was measured by the Brier score DT: 0.19, Naïve Bayes: 0.23, SVM: 0.23, RF: 0.22, GB: 0.22, MLP: 0.33	Cross-validation (five-fold cross-validation)	Anaconda3-5.1.0-Windows Scikit-learn v0.19.1 for implementing LR, DT, random forest, Naive Bayes, SVM, and gradient boosting models Keras v2.2.4 for implementing MLP models
Zack *et al.*^56^	RF regression model	LR	410 variables for the CHF readmission and long-term mortality models; 52 variables were used for in-hospital mortality models	Used multiple imputation techniques Applied imputation by predictive mean matching Ensured the same imputation model was used across all data sets Conducted analysis on both original and imputed data sets	NR	ROC AUC: provided as *C*-statistic Other metrics: calibration comparing observed and expected event rates in deciles of risk	Exploratory data analysis: not explicitly mentioned, though cross-validation methodology was used for internal validation	All-cause in-hospital mortality (death in hospital or within 3 days of discharge): 0.92 180-day all-cause mortality: 0.87 180-day cardiovascular mortality: 0.88 180-day heart failure related mortality: 0.90 30-day CHF readmission: 0.90	No mention of calibration	Cross-validation (eight-fold cross-validation)	Python with Scikit-learn for model development
Zhao *et al.*^57^	Extreme Gradient Boosting (XGBoost)	CRUSADE ACUITY-HORIZONS	98 variables	Sparsity-Aware Algorithm Automatic feature selection validation and stability checks Consistency across data sets	70% training and 30% testing	ROC AUC: provided as *C*-statistic Sensitivity/specificity PPV/NPV Accuracy Other metrics: calibration curves	NR	Derivation data set: 0.941 (0.909–0.973, *P* < 0.001) Validation data set: 0.837 (0.772–0.903, *P* < 0.001)	XGBoost was calibrated using the quintile plot of observed vs. predicted risk and Hosmer–Lemeshow goodness-of-fit test (11.507, *P* = 0.201). This suggested that the XGBoost model on the validation data set suggested no evidence of lack of fit (*P* > 0.05)	Single random split (70% training and 30% testing)	Extreme Gradient Boosting (XGBoost)

AMI, acute myocardial infarction; DT, decision tree; LR, logistic regression; GAM, generalized additive model; RF, random forest; GB, gradient boosted tree; NN, neural network; NCDR, National Cardiovascular Data Registry; MLP, multi-layer perceptron; NPV, negative predictive value; PPV, positive predictive value; PRAISE, Prediction of Adverse Events Following an Acute Coronary Syndrome.

**Table 3:** Baseline variables used in the machine learning models

Variables	Study												
	Călburean *et al*.^48^	Chao *et al.*^58^	Doll *et al.*^2^	Li *et al.*^49^	Mortazavi *et al.*^50^	Niimi *et al.*^51^	Rayfield *et al.*^59^	Resnic *et al.*^52^	Sampedro *et al.*^53^	Shi *et al.*^54^	Xiao *et al.*^55^	Zack *et al.*^56^	Zhao *et al.*^57^
Demographics													
Age	x	x	x	x	x	x	x	x	x	x	x	x	x
Gender (sex)	x	x	x	x	x	x	x	x	x	x	x	x	x
Smoking status	x	x	x	x	x		x		x		x	x	x
Body mass index				x	x	x	x		x	x	x	x	x
Blood pressure				x	x	x	x		x	x	x	x	x
Comorbidities and past medical history													
Diabetes mellitus	x	x	x	x	x	x	x	x	x	x	x	x	x
Hypertension		x	x	x	x	x	x		x	x	x	x	x
Dyslipidaemia				x	x	x	x		x	x		x	x
Coronary artery disease				x	x		x		x		x	x	x
Previous myocardial infarction			x	x	x		x	x	x	x		x	x
Heart failure (history or within 2 weeks)	x		x	x	x	x	x	x	x			x	x
Chronic kidney disease (or estimated glomerular filtration rate)			x	x	x	x	x	x	x	x	x	x	x
Peripheral artery disease				x	x		x		x	x		x	x
Atrial fibrillation					x		x		x			x	x
Chronic obstructive pulmonary disease			x		x		x		x	x		x	x
Stroke/transient ischaemic attack			x	x	x		x		x	x	x	x	x
Previous cardiac surgery/intervention			x	x	x	x	x			x	x	x	x
Oncological disease					x		x		x			x	x
New York Heart Association (NYHA) functional class					x		x	x				x	x
Medication usage (e.g. beta-blockers, Angiotensin Converting Enzyme (ACE) inhibitors)				x	x		x		x	x	x	x	x
Cardiogenic shock at presentation		x	x		x	x	x	x		x	x	x	x
ST-elevation myocardial infarction	x	x	x		x	x	x	x		x	x	x	x
Non-ST-elevation acute coronary syndrome	x	x	x		x	x	x			x	x	x	x
Use of intra-aortic balloon pump		x			x	x	x					x	x
Biomarkers and laboratory values													
Creatinine levels		x	x	x	x	x	x			x	x	x	x
Haemoglobin levels		x	x	x	x	x	x			x		x	x
Albumin levels					x		x					x	x
Troponins			x	x	x		x					x	x
International normalized ratio		x	x		x		x					x	x
Platelet count							x				x	x	x
Echocardiography													
Left ventricular ejection fraction	x		x	x	x		x			x	x	x	x
Aortic valve gradient					x								
Vmax					x								
Aortic valve area					x								
Mitral regurgitation				x	x		x						x
Angiographic characteristics													
Lesion complexity			x	x	x		x			x	x	x	x
Stenosis %			x	x	x		x			x	x	x	x
Number of diseased vessels				x	x		x			x	x	x	x
Thromoblysis in Myocardial Infarction (TIMI) flow grade	x				x		x			x	x	x	x
Presence of Left Main Coronary Artery disease					x		x			x		x	x

*Table 2* summarizes the methods of validation employed across each study. Single random split (70% training and 30% test) was the most common, used in 23.1% of the studies. Similarly, 23.1% used five-fold cross-validation, where the data set is divided into five parts, and each part is tested once. External validation with a chronological split and 10-fold cross-validation were each used in 7.7% of the studies. Other methods, each used in 7.7% of the studies, included single random splits into training and test sets, 10-fold cross-validation with nested cross-validation for hyper-parameter tuning, the X-tile method for cohort splitting, and eight-fold cross-validation.

### Methodological quality and risk of bias

Using the PROBAST, all top-performing models demonstrated an overall high risk of bias (see Supplementary material for a detailed risk of bias breakdown). Analysis was of greatest concern due to poor reporting of calibration statistics, predictor weighting, and inappropriate handling of missing or complex data. This was due to elevated risks observed in the events per variable subsection (80.0%) and the missing data subsection (50.0%). Only three studies (23.1%) adhered to the TRIPOD statement, with two studies providing a checklist in the Supplementary material. Concerning the transparency of models and data-sharing practices, five studies (38.5%) included data availability statements, specifying that data would be made accessible upon reasonable request, whereas one study (7.7%) explicitly stated that data could not be shared. Two studies (15.4%) provided the statistical code utilized in model development and assessment; from the remaining studies, specific hypertuning parameters were only available in two studies (15.4%). Additionally, one study (7.7%) shared an anonymized data set for external validation. A complete risk of bias assessment is detailed in the Supplementary material.

### Model evaluation

The *C*-statistic (area under the ROC curve) was the predominant model performance evaluation metric, reported in 100% of studies. Sensitivity and specificity were also commonly used, appearing in 53.8% of the studies. Other metrics included precision-recall curve (PR)-AUC (38.5%), accuracy (30.8%), and positive predictive value/negative predictive value (38.5%). F1 score was reported in 15.4% of the studies, while Matthews correlation coefficient (MCC) and net reclassification improvement (NRI) were mentioned in select studies as additional performance measures. For calibration, 53.8% of the studies employed calibration plots, making it the most frequent method, followed by the Brier score (38.5%) and the Hosmer–Lemeshow test (23.1%). Model evaluation metrics are summarized for each study in *Table 2*.

### All-cause mortality

**Figure 3:**
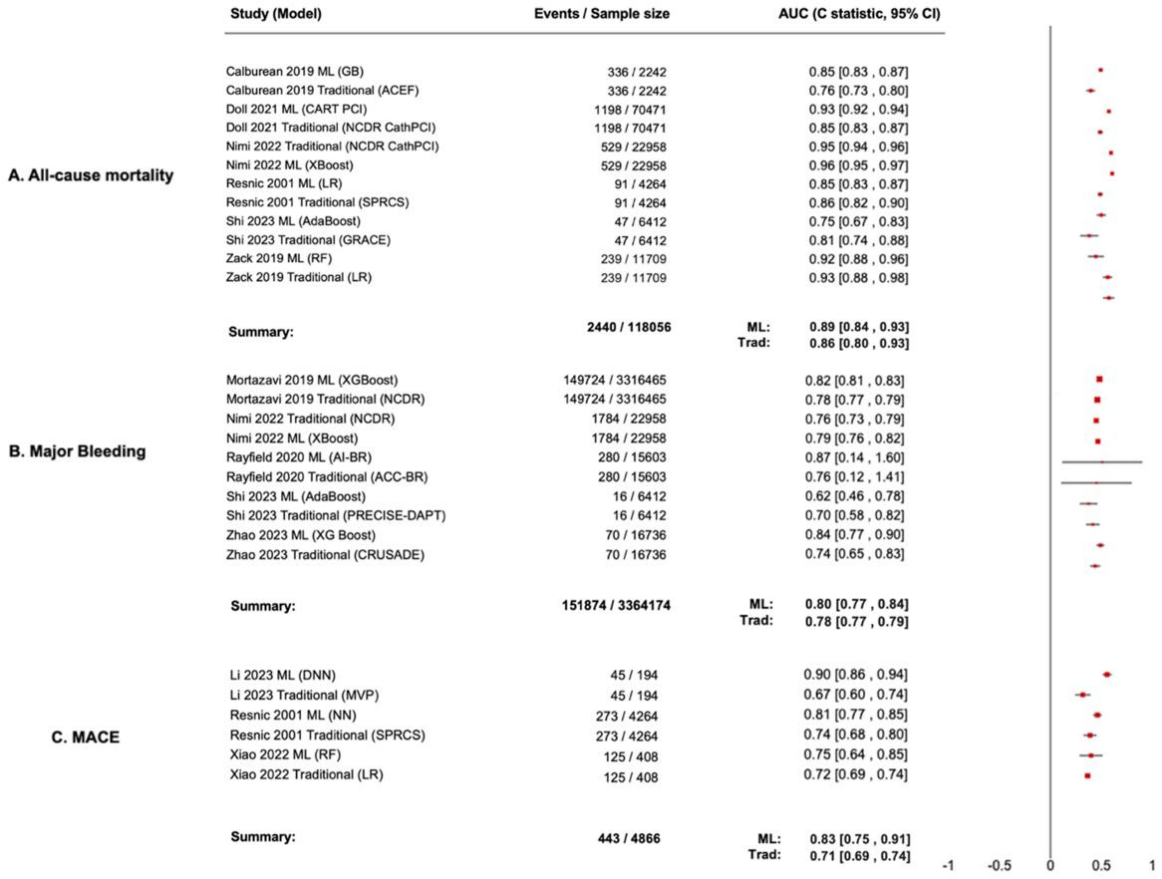
Meta-analysis of predictive performance of machine learning models. Forest plot of outcome-specific *C*-statistics (95% confidence intervals) stratified according to outcomes representing (*A*) all-cause mortality, (*B*) major bleeding, and (*C*) major adverse cardiovascular events.

Six studies were aimed at prediction of all-cause mortality after PCI. The reported time points for all-cause mortality varied: 30-day mortality post-PCI (two studies), in-hospital mortality (three studies), and 3-year all-cause mortality (one study). As shown in *Figure 3*, the pooled *C*-statistic of the top-performing ML models for all-cause mortality was 0.89 (95% CI, 0.84–0.91). This was superior to the pooled traditional risk scores *C*-statistic for all-cause mortality: 0.86 (95% CI, 0.80–0.93), but did not demonstrate a statistically significant difference in *C*-statistic (*P* = 0.54). Discriminative performance of ML models compared with traditional risk scores (*C*-statistic with 95% CIs and prediction intervals) for all-cause mortality is summarized in *Table 4*.

**Table 4:** Discriminative performance of machine learning models compared with traditional risk scores (*C*-statistic with 95% confidence intervals and prediction intervals)

Study	*C*-statistic (95% CI)	Prediction interval
All-cause mortality traditional methods		
Călburean *et al.*^48^ traditional (ACEF)	0.76 (0.73–0.80)	(0.70, 0.82)
Doll *et al.*^2^ traditional (NCDR-Cath PCI)	0.85 (0.83–0.87)	(0.82, 0.88)
Niimi *et al.*^51^ traditional (NCDR-Cath PCI)	0.95 (0.94–0.96)	(0.93, 0.97)
Resnic *et al.*^52^ traditional Society for Cardiovascular Angiography and Interventions (SCAI) Percutaneous Coronary Intervention Risk Stratification score (SPRCS)	0.86 (0.82–0.90)	(0.72, 1.00)
Shi *et al.*^54^ traditional (GRACE)	0.81 (0.84–0.88)	(0.69, 0.93)
Zack *et al.*^56^ traditional (LR)	0.93 (0.88–0.98)	(0.88, 0.98)
All-cause mortality ML methods		
Călburean *et al.*^48^ ML (GB)	0.85 (0.83–0.87)	(0.82, 0.88)
Doll *et al.*^2^ ML (CART PCI)	0.93 (0.92–0.94)	(0.91, 0.95)
Niimi *et al.*^51^ ML (XBoost)	0.96 (0.95–0.97)	(0.94, 0.98)
Resnic *et al.*^52^ ML (LR)	0.85 (0.83–0.87)	(0.82, 0.88)
Shi *et al.*^54^ ML (AdaBoost)	0.75 (0.67–0.83)	(0.61, 0.89)
Zack *et al.*^56^ ML (RF)	0.92 (0.88–0.96)	(0.85, 0.99)
Major bleeding traditional methods		
Mortazavi *et al.*^50^ traditional (NCDR)	0.78 (0.77–0.79)	(0.76, 0.80)
Niimi *et al.*^51^ traditional (NCDR)	0.76 (0.73–0.79)	(0.70, 0.82)
Rayfield *et al.*^59^ traditional American College of Cardiology Bleeding Risk Score (ACC-BR)	0.764 (0.12–1.41)	(−0.53, 2.06)
Shi *et al.*^54^ traditional Predicting Bleeding Complications in Patients Undergoing Dual Antiplatelet Therapy score (PRECISE-DAPT)	0.7 (0.58–0.82)	(0.46, 0.94)
Zhao 2023^57^ traditional (CRUSADE)	0.74 (0.65–0.83)	(0.57, 0.91)
Major bleeding ML methods		
Mortazavi *et al.*^50^ ML (XGBoost)	0.82 (0.81–0.83)	(0.80, 0.84)
Niimi *et al.*^51^ ML (XBoost)	0.79 (0.76–0.82)	(0.73, 0.85)
Rayfield *et al.*^59^ ML (AI-BR)	0.87 (0.14–1.60)	(−0.60, 2.34)
Shi *et al.*^54^ ML (AdaBoost)	0.62 (0.46–0.78)	(0.30, 0.94)
Zhao 2023^57^ ML (XG Boost)	0.84 (0.77–0.90)	(0.71, 0.97)
MACE traditional methods		
Li *et al.*^49^ traditional microvascular perfusion (MVP)	0.67 (0.60–0.74)	(0.33, 1.01)
Resnic *et al.*^52^ traditional (SPRCS)	0.74 (0.68–0.80)	(0.45, 1.03)
Xiao *et al.*^55^ traditional (LR)	0.72 (0.69–0.74)	(0.32, 1.12)
MACE ML methods		
Li *et al.*^49^ ML Deep Neural Network (DNN)	0.90 (0.86–0.94)	(0.71, 1.09)
Resnic *et al.*^52^ ML (NN)	0.81 (0.77–0.85)	(0.54, 1.08)
Xiao *et al.*^55^ ML (RF)	0.75 (0.64–0.85)	(0.24, 1.26)

ACC-BR, American College of Cardiology-Cath PCI Bleeding Risk.

### Major bleeding

Major bleeding outcomes were reported in five out of the 13 included studies (38.5%). Different definitions and time points were used: in-hospital major bleeding within 72 h post-PCI (one study), post-PCI bleeding within 72 h and prior to hospital discharge (one study), major bleeding (Bleeding Academic Research Consortium [BARC] Type 3 or 5) at 1 year (one study), and in-hospital major bleeding (BARC Type 3 or 5 of non-coronary artery bypass grafting)-related bleeding) (one study). The pooled *C*-statistic for top-performing ML models for major bleeding was 0.80 (95% CI, 0.77–0.84), compared with traditional methods 0.78 (95% CI, 0.77–0.79). The difference in *C*-statistic was 0.02 (*P* = 0.02) (*Figure 3*). Discriminative performance of ML models compared with traditional risk scores (*C*-statistic with 95% CIs and prediction intervals) for major bleeding is summarized in *Table 4*.

### Major adverse cardiovascular events

Major adverse cardiovascular events were reported in three out of the 13 included studies (23.1%). Definitions included cardiac death, hospitalization for congestive heart failure, re-infarction, stroke, and recurrent angina (one study); combined endpoints of in-hospital death, myocardial infarction, and same stay bypass surgery (one study); and myocardial infarction, heart failure, kidney failure, coronary events, cerebrovascular events, and death (one study). The *C*-statistic for top-performing ML models for MACE was 0.83 (95% CI, 0.75–0.91) vs. traditional methods 0.71 (95% CI, 0.69–0.74). The difference in *C*-statistic was 0.12 (*P* = 0.007) (*Figure 3*). Discriminative performance of ML models compared with traditional risk scores (*C*-statistic with 95% CIs and prediction intervals) for MACE is summarized in *Table 4*.

## Discussion

### Summary of findings

This systematic review and meta-analysis compared discrimination between ML methods and traditional approaches for prediction of clinical outcomes following PCI. Machine learning models marginally outperformed traditional risk scores in the discrimination of major bleeding events and the composite outcome MACE following PCI. Notably, only one ML algorithm had completed external validation, and the included studies exhibited a high level of bias, along with minimal reporting of calibration and risk reclassification measures. See *figure 1* for central graphical abstract summary.

### Comparison with previous studies

Machine learning algorithms have demonstrated inconsistent results when compared with traditional statistical methods.^21,30–34^ While no systematic reviews have directly compared ML and standard regression or other traditional methods for clinical outcome prediction after PCI, Benedetto *et al.*^22^ found incremental advantage of ML in mortality prediction post-cardiac surgery and Liu *et al.*^21^ demonstrated moderate improvement in prognostication of atherosclerotic cardiovascular risk. Conversely, Christodoulou *et al.*^23^ observed no difference in predictive accuracy between ML and regression in low-bias studies but did find a difference in studies with high bias. Notably, all studies included in the current study exhibited a high risk of bias.

### Model performance: strengths

The present findings support the hypothesis that ML models can achieve superior discrimination in the prediction of adverse clinical outcomes after PCI. The models evaluated in this study incorporate common variables that are evaluable before PCI and can be readily obtained from electronic health records (EHRs). It is worth noting that although the inclusion of additional procedural variables could enhance model performance, it may reduce their utility as point-of-care tools. For studies that included traditional risk scores in their ML model feature set and were trained on the same registry, there was a significant net reclassification improvement for nearly all endpoints. The ML models included in this review are derived from objective data accessible to clinicians before angiography and PCI or to the patient’s care team before hospital discharge, depending on the clinical endpoint. This pre-procedural and peri-discharge availability of data supports timely and informed decision-making. Many of the included covariates in the final ML models and their distribution across studies as presented in this review have been previously demonstrated to be independent predictors of the risk of in-hospital adverse outcomes.^60,61^ Other models incorporated large magnitude of data and all available variables (up to 410 variables) many of which are unlikely to be included in traditional LR models.

One potential advantage of ML models is that they can incorporate disparate data types and seamlessly integrate into EHRs. For example, ML models can utilize both conventional parameters (e.g. a numerical field such as systolic blood pressure) and parameters in other forms, such as imaging. It is possible to combine such parameters in a given ML model to predict outcomes such as MACE.^62^ Advanced ML techniques, such as neural networks, have demonstrated excellent performance in handling high-dimensional and highly self-correlated data, such as medical imaging, which are beyond the capabilities of classic statistical models^63^ This is pertinent to the studies included in this review, as 69.2% of included ML models specified echocardiographic parameters (see *Table 3*). Furthermore, compared with traditional statistical methods, ML algorithms efficiently handle missing data as they do not rely on data distribution assumptions and are capable of more complex calculations. Incorporating risk scores into EHRs could redefine their evaluation criteria. By aligning risk scores with discrete data elements from EHRs, they can be seamlessly integrated into clinical decision support systems, enabling automatic calculation for relevant patients.^64^ Unlike classical regression models that require static framework and set predictors, ML models can discern and analyse complex, non-linear variable interactions for more nuanced risk assessments, potentially uncovering relationships not evident in linear models,^65^ enabling targeted interventions to decrease mortality and readmission.^56,66–68^ Artificial neural networks were expected to provide the greatest discriminatory power because they can model non-linearly separable relationships, unlike linear methods such as LR.^69^ However, it is important to note that significant improvements with ML models were observed only when the best ML model from each study was selected, pooling different types of ML algorithms. When focusing on individual ML model categories, we hypothesized that these improvements would not persist, supporting the No Free Lunch theorem in ML.^70^ This theorem posits that no single model works best for every problem or data set, emphasizing the necessity to try multiple models to find the most suitable one for a specific problem. We attempted to determine discrimination of ML models according to sub-type; however, the discrepancy in the number of ML models available for each outcome precluded any meaningful subgroup analysis. Therefore, the magnitude and clinical influence of ML model improvements remain uncertain.

### Model performance: weaknesses

In the current review, included studies comparing ML algorithms with traditional risk scores exhibited significant weaknesses. Most studies utilized a retrospective cohort design; only one of 13 studies provided external validation, with the majority assessed as having a high risk of bias. While calibration was reported in 10 studies, signifying a comparative improvement from previous studies, the lack of uniformity in calibration metrics highlights a deficiency in reporting standards.^21–23,30,46,71,72^ Three studies followed the TRIPOD reporting statement, reflecting limited uptake in artificial intelligence (AI) research and the need for an updated guideline.^73,74^ Furthermore, there was limited evaluation of model accuracy in a real-world scenario through an externally validated, prospective cohort study design.

Variations in patient demographics, risk factors, and therapeutic approaches significantly influence cardiovascular burden, clinical outcomes, care quality, and the predictive accuracy of comparable scores. Numerous studies rely on large single-centre registries with well-structured data sets that integrate EHR data, patient-reported outcomes, and dedicated research personnel. Effective application of these models necessitates meticulous data collection with immediate electronic availability, including documentation that is currently recorded in physician notes. The reviewed models were capable of managing small amounts of missing data but proved unreliable with larger deficits. Most studies employed multiple imputation techniques to address missing data and conducted sensitivity and robustness analyses. Some studies opted to exclude patients with substantial missing data or variables with high rates of missingness. Integrating these models for automated computation within EHR systems demands considerable clinical and IT resources.

A recent state-of-the-art review highlighted that many cardiovascular clinical prediction models in the literature lack external validation, often resulting in performance that appears overly optimistic due to a reduction in validation during model development phases.^63^ Single external validations are insufficient to fully grasp performance variability across different scenarios. Machine learning modelling necessitates a higher number of events per variable to achieve a stable *C*-statistic compared with LR, making it suitable only for very large data sets.^75^ Both ML and LR models perform inadequately with small data sets or low event occurrences. Machine learning algorithms may generate unsatisfactory classifiers when faced with imbalanced data sets, favouring the majority class and achieving high accuracy but misclassifying the minority class, known as the accuracy paradox.^76^ To address class imbalance, over- and under-sampling or algorithm modifications can be employed. This challenge is particularly relevant for patients undergoing PCI due to the low incidence of adverse events and the variability in ML model feature selection across studies. For LR models, unbalanced training data impact only the model intercept, which can be corrected. Moreover, traditional risk models are developed using structured data sets (i.e. global registry of acute coronary events [GRACE] score or can rapid risk stratification of unstable angina patients suppress ADverse outcomes with early implementation of the ACC/AHA guidelines [CRUSADE] score), which contain only a restricted number of prespecified variables and thus, limiting the capability of ML, which may perform best by exploiting high-dimensional data from electronic medical records. Beyond performance measurement, deploying and maintaining ML-based risk models is challenging due to their lack of explainability and risk of overfitting, whereas traditional LR models like the NCDR risk scores are easier to implement and update.

Critical appraisal of ML-based prediction models includes assessing the necessity of new models, their integration into clinical workflows, data representativeness, appropriate feature measurement intervals (pre- or post-PCI), and outcome reliability. Other key evaluation factors include sample sizes, thoroughness of internal and external validation, performance beyond classification, adherence to reporting guidelines, algorithmic fairness, model openness for external use and validation, and detailed interpretation of feature–outcome relationships. These criteria aim to uphold scientific rigour in ML research. Our findings are concerning: a high risk of bias was noted in most studies, data-sharing practices lacked transparency, and few studies adhered to guidelines.

### Clinical application

Accurate risk prediction enables personalized informed consent and appropriate preparation for high-risk procedures. Experienced PCI operators and hospitals have reported increasing success rates and lower complication rates among left main, multi-vessel, chronic total occlusion, and other high-risk or complex patients. Early identification of these patients allows for team-based preparation and early risk mitigation strategies. For instance, patients at risk for heart failure readmission can receive intensive education and early follow-up to reduce readmission rates efficiently. However, before developing a new ML or any prediction model, it is essential to question its necessity. Given the numerous existing risk stratification models, particularly for mortality prediction, the need for creating additional models may be questionable. Current PCI risk stratification models are limited by their reliance on procedural data, narrow outcome focus, and insufficient prognostic ability.^4,8,9,77–79^ The *ad hoc* nature of most PCI procedures conducted during diagnostic angiography undermines the utility of risk models dependent on angiographic characteristics for risk assessment.^5,78,80^ Despite the efficacy of various risk scores, their integration into clinical practice remains limited. A retrospective study indicated that only 57% of patients in the Netherlands with non-ST-segment elevation acute coronary syndromes had a risk score assessed.^15^ Furthermore, there is an absence of data regarding the proportion of cardiologists employing PCI-specific risk scores.^81^ Factors contributing to this underutilization, as identified in a qualitative study, include unawareness of the risk scores, time constraints, and the perceived irrelevance of these scores. This situation mirrors a ‘last mile’ problem in supply chain management, where despite the availability of effective risk prediction tools, they fail to be adequately implemented in clinical setting. Although traditional bleeding risk scores have been intended to guide antithrombotic therapy after PCI, low contemporary uptake suggests clinicians are less likely to apply these risk scores without established thresholds proven to guide clinical decision-making and outcomes.

A hypothesized benefit of ML models is the ability to continuously improve predictive accuracy by assimilating new data in real time, without needing daily input from research staff or a statistician. Linked to EHRs, these models can automatically calculate risk scores at the time of PCI or before discharge, eliminating manual calculations and potentially increasing clinical use. High-risk patient subgroups identified through these models can receive targeted preventative care cost-efficiently. While classical regression models can also be updated with new data, they require fixed input predictors and functional forms. Ultimately, although ML approaches enable the analysis of large data sets, identification of new associations, and improvement in previous prognostic models and diagnostic accuracy, its direct clinical application for patients undergoing PCI is insufficient to translate into meaningful clinical changes, such as improved discrimination and accuracy in risk prediction.

### Recommendations

Despite high expectations for AI in medicine, translating ML models from EHRs to real-life clinical practice has faced obstacles. Few peer-reviewed ML models have been routinely implemented in hospitals, and a recent study identified only 21 ML models applied in healthcare settings.^24^ The gap between model development and deployment arises from model flaws, logistical challenges, lack of multi-disciplinary co-operation, insufficient implementation infrastructure, privacy and sociocultural issues, and the need for ongoing model assessment and financial support. Some authors compare translational ML with drug discovery, requiring further validation in clinical trials. While we agree with previous authors that external validation of data is necessary, it should be conducted by independent authors where feasible to avoid publication and reporting bias.

Calibration for all models is recommended, with reliability graphs and calibration metrics to detect agreement between predicted and actual values. A recent review found that nearly 95% of ML-based prediction models lacked calibration data despite guidelines recommending it. Calibration is crucial for real-world application, as poorly calibrated models can lead to misleading predictions. For example, over-estimating mortality risk may inappropriately dissuade a patient from undergoing a procedure. Where over-estimation occurred, a plausible mechanism may be the low event rates observed in included studies. Imbalanced data challenge ML models, as they often prioritize the majority class over the minority class, leading to biased calibration performance. The *C*-statistic or AUC-ROC was chosen as preferred measure of model performance due to its intuitiveness, ability to differentiate cases and non-cases, and uniform reporting across all studies, but it has significant limitations, including no measure of calibration, predictive accuracy, and magnitude of risk difference. For all future studies, uniform reporting standards and performance metrics are essential for consistent evaluations and comparisons, promoting the development of robust prediction models and reliable meta-analysis. Additionally, hospital readmissions after PCI have previously been shown to be predominantly non-cardiac, with few 30-day events related to the index PCI.^82^ Machine learning’s ability to integrate extensive patient-level data from EHRs should improve the prediction of non-cardiac events and mortality after PCI, and future studies should focus on identifying patients at high risk for non-cardiac mortality or readmission.

Our findings of low adherence to TRIPOD guidelines highlight the need for rigorous review of current ML research practices. One reason may be that the statement’s design does not adequately address AI models, posing applicability challenges. To address this, the TRIPOD-AI statement^83^ has been developed to help researchers design studies and reduce systematic bias. Alternatively, the unmet criteria in our review may indicate inconsistent research practices. Early adopters of AI/ML technologies must ensure scientific reliability and validity through collaboration among clinicians, data scientists, and statisticians.

High-quality evidence is the cornerstone of clinical integration, yet the benchmark for such evidence in ML applications remains nebulous. The risk–benefit balance of adopting ML technologies early (potentially introducing inefficiencies) vs. delaying their implementation (possibly forgoing early patient benefits) is currently the subject of ongoing discussion.^84^ The financial model for ML technologies, particularly post-development, is an area of active exploration.^26,85^ An oft-cited hurdle is the perceived ‘black box’ nature of ML models, which some argue could hinder clinical adoption^86,87^ The solution may lie in establishing standardized guidelines and performance metrics that promote seamless integration and expedited development. These studies highlight the pressing requirement for further external validation studies and standardized reporting guidelines in AI research.^88^ Robust external validation with data sets from diverse centres is necessary to progress such algorithms to the point of implementation and associated potential improvement in patient outcomes. International collaborations that promote secure data sharing and algorithm exchange facilitate such validation. In addition, existing research regulatory systems should support such research, with informed and proactive approaches promoted by institutional review boards. Rather than seeking a one-size-fits-all ML algorithm, customizing adaptive models for specific data sets may be more effective.^21–24,89^ Implementation faces significant hurdles, including data governance and the requirement for regulatory compliance.^21,24,90^

### Limitations

Consistent with previous ML literature, this review exhibits considerable limitations.^21,30–34^ First, while we have outlined the variety of ML models used, we are unable to elucidate the specific reasons behind the choice of algorithms for predicting outcomes in patients undergoing PCI. Additionally, our categorization of model performance, based on predefined literature thresholds, lacks a detailed justification for these benchmarks. A significant limitation is our study’s inability to assess the clinical relevance of the features selected or their impact on the model’s predictive accuracy, due to a lack of prospective externally validated studies. The disclosure of ML algorithm design details is often inadequate, with a lack of information on neural network architectures, training durations, data availability, and hyper-parameters.^2,21,48–59,89^ Heterogeneity in ML model training also creates challenges in interpretability, and variability in internal validation, baseline demographics, covariates, and metric reporting further complicate model evaluation.^91–93^ The wide prediction intervals calculated for ML models may reflect significant variability in the standard errors and highlight the influence of each study’s precision on the interval width. These intervals provide an estimate of where the true *C*-statistic for future studies is likely to fall, considering both the accuracy and variation between studies. The scarcity of externally validated models hinders the assessment of their true predictive power and the risk of overfitting.^21,89^ As such, the precise utility of ML in risk prediction following PCI is yet to be established. As such, despite growing research interest due to its potential for innovation, ML has not been formally recommended in clinical risk stratification guidelines.^26^

## Conclusions

This meta-analysis found that ML models marginally outperformed traditional risk scores in the discrimination of MACE and major bleeding events following PCI. While integration of ML algorithms into electronic healthcare systems may play a role in future peri-procedural risk stratification, immediate implementation in the clinical setting remains uncertain. Further research is required to overcome methodological and validation limitations.

## Supplementary Material

ztae074_Supplementary_Data

## Data Availability

The data underlying this article are available in the article and in its online Supplementary material.
