# Supplementary material for: Machine learning approaches for risk prediction after percutaneous coronary intervention: a systematic review and meta-analysis
Source: Eur Heart J Digit Health. 2024 Oct 14;6(1):23–44. doi: 10.1093/ehjdh/ztae074 (PMC11750198; doi:10.1093/ehjdh/ztae074)
Supplement: ztae074_Supplementary_Data [file ztae074_supplementary_data.docx]

**SUPPLEMENTARY DIGITAL CONTENT**

**List of Supplementary Digital Content**

- Appendix 1: PRISMA 2020 Checklist
- Appendix 2: TRIPOD and CHARMS checklist
- Appendix 3: Search String
- Appendix 4: Justification of exclusions at full-text review
- Appendix 5: Risk of bias assessment for included studies using the PROBAST risk of bias tool

**Appendix 1:**

**Supplementary Table 1 - PRISMA 2020 Checklist**

| **Section and Topic** | **Item #** | **Checklist item** | **Location where item is reported** |
| --- | --- | --- | --- |
| **TITLE** | | |  |
| Title | 1 | Identify the report as a systematic review. | Title page |
| **ABSTRACT** | | |  |
| Abstract | 2 | See the PRISMA 2020 for Abstracts checklist. | 2 |
| **INTRODUCTION** | | |  |
| Rationale | 3 | Describe the rationale for the review in the context of existing knowledge. | 3-4 |
| Objectives | 4 | Provide an explicit statement of the objective(s) or question(s) the review addresses. | 3-4 |
| **METHODS** | | |  |
| Eligibility criteria | 5 | Specify the inclusion and exclusion criteria for the review and how studies were grouped for the syntheses. | 4, 19 (Figure 1) |
| Information sources | 6 | Specify all databases, registers, websites, organisations, reference lists and other sources searched or consulted to identify studies. Specify the date when each source was last searched or consulted. | 4 |
| Search strategy | 7 | Present the full search strategies for all databases, registers and websites, including any filters and limits used. | 4, Supp. p.8 |
| Selection process | 8 | Specify the methods used to decide whether a study met the inclusion criteria of the review, including how many reviewers screened each record and each report retrieved, whether they worked independently, and if applicable, details of automation tools used in the process. | 4 |
| Data collection process | 9 | Specify the methods used to collect data from reports, including how many reviewers collected data from each report, whether they worked independently, any processes for obtaining or confirming data from study investigators, and if applicable, details of automation tools used in the process. | 4-5 |
| Data items | 10a | List and define all outcomes for which data were sought. Specify whether all results that were compatible with each outcome domain in each study were sought (e.g. for all measures, time points, analyses), and if not, the methods used to decide which results to collect. | 4-7 |
|  | 10b | List and define all other variables for which data were sought (e.g. participant and intervention characteristics, funding sources). Describe any assumptions made about any missing or unclear information. | 4-7 |
| Study risk of bias assessment | 11 | Specify the methods used to assess risk of bias in the included studies, including details of the tool(s) used, how many reviewers assessed each study and whether they worked independently, and if applicable, details of automation tools used in the process. | 6 |
| Effect measures | 12 | Specify for each outcome the effect measure(s) (e.g. risk ratio, mean difference) used in the synthesis or presentation of results. | 5 |
| Synthesis methods | 13a | Describe the processes used to decide which studies were eligible for each synthesis (e.g. tabulating the study intervention characteristics and comparing against the planned groups for each synthesis (item #5)). | 4-6 |
|  | 13b | Describe any methods required to prepare the data for presentation or synthesis, such as handling of missing summary statistics, or data conversions. | 4-7 |
|  | 13c | Describe any methods used to tabulate or visually display results of individual studies and syntheses. | 5-7 |
|  | 13d | Describe any methods used to synthesize results and provide a rationale for the choice(s). If meta-analysis was performed, describe the model(s), method(s) to identify the presence and extent of statistical heterogeneity, and software package(s) used. | 6-7 |
|  | 13e | Describe any methods used to explore possible causes of heterogeneity among study results (e.g. subgroup analysis, meta-regression). | 6-7 |
|  | 13f | Describe any sensitivity analyses conducted to assess robustness of the synthesized results. | 5 |
| Reporting bias assessment | 14 | Describe any methods used to assess risk of bias due to missing results in a synthesis (arising from reporting biases). | 6-7 |
| Certainty assessment | 15 | Describe any methods used to assess certainty (or confidence) in the body of evidence for an outcome. | 2, 5 |
| **RESULTS** | | |  |
| Study selection | 16a | Describe the results of the search and selection process, from the number of records identified in the search to the number of studies included in the review, ideally using a flow diagram. | 6, 19 (Figure 1) |
|  | 16b | Cite studies that might appear to meet the inclusion criteria, but which were excluded, and explain why they were excluded. | 19 (Figure 1), Supp. p.11-16 |
| Study characteristics | 17 | Cite each included study and present its characteristics. | 25-28 (Table 1) |
| Risk of bias in studies | 18 | Present assessments of risk of bias for each included study. | 6, Supp. p26 |
| Results of individual studies | 19 | For all outcomes, present, for each study: (a) summary statistics for each group (where appropriate) and (b) an effect estimate and its precision (e.g. confidence/credible interval), ideally using structured tables or plots. | 7-8, 20-21 (Figure 2 and 3) |
| Results of syntheses | 20a | For each synthesis, briefly summarise the characteristics and risk of bias among contributing studies. | 6-7, Supp. p16 |
|  | 20b | Present results of all statistical syntheses conducted. If meta-analysis was done, present for each the summary estimate and its precision (e.g. confidence/credible interval) and measures of statistical heterogeneity. If comparing groups, describe the direction of the effect. | 20-24 |
|  | 20c | Present results of all investigations of possible causes of heterogeneity among study results. | 5 (heterogeneity was not formally assessed and assumed to be present) |
|  | 20d | Present results of all sensitivity analyses conducted to assess the robustness of the synthesized results. | 4-5 |
| Reporting biases | 21 | Present assessments of risk of bias due to missing results (arising from reporting biases) for each synthesis assessed. | 6-7, Supp. p16 |
| Certainty of evidence | 22 | Present assessments of certainty (or confidence) in the body of evidence for each outcome assessed. | 5-7 20-24, Figure 2-6 |
| **DISCUSSION** | | |  |
| Discussion | 23a | Provide a general interpretation of the results in the context of other evidence. | 9-10 |
|  | 23b | Discuss any limitations of the evidence included in the review. | 9-10 |
|  | 23c | Discuss any limitations of the review processes used. | 9-10 |
|  | 23d | Discuss implications of the results for practice, policy, and future research. | 9-10 |
| **OTHER INFORMATION** | | |  |
| Registration and protocol | 24a | Provide registration information for the review, including register name and registration number, or state that the review was not registered. | 4 |
|  | 24b | Indicate where the review protocol can be accessed, or state that a protocol was not prepared. | 4 |
|  | 24c | Describe and explain any amendments to information provided at registration or in the protocol. | Not applicable |
| Support | 25 | Describe sources of financial or non-financial support for the review, and the role of the funders or sponsors in the review. | Title page |
| Competing interests | 26 | Declare any competing interests of review authors. | Title page |
| Availability of data, code and other materials | 27 | Report which of the following are publicly available and where they can be found: template data collection forms; data extracted from included studies; data used for all analyses; analytic code; any other materials used in the review. | Title page |

**Appendix 2**

**TRIPOD-SRMA checklist**

| **Section and**  **topic** | **Item**  **No** | **Checklist item** | **Page** |
| --- | --- | --- | --- |
| **Title** |  |  |  |
| Title | 1 | Identify the report as a systematic review or meta-analysis (or both) of diagnostic or prognostic model studies. Specify the target population and  outcome(s) predicted as relevant to the review question. | Title Page, 5 |
| **Abstract** |  |  |  |
| Abstract | 2 | See the TRIPOD-SRMA Checklist for Abstracts | 2 |
| **Introduction** |  |  |  |
| Rationale | 3 | Describe the rationale for the review in the context of existing knowledge. | 3-4 |
| Objectives | 4 | Provide an explicit statement of the objective(s) being addressed with reference to: target population, index and comparator models (as relevant),  outcome(s), time (prediction horizon and intended moment of using the model), and setting. | 3-4 |
| **Methods** |  |  |  |
| Study eligibility  criteria | 5 | Specify study characteristics used as eligibility criteria, including any prediction models of specific interest, and whether development or validation  studies (or both) were eligible. | 5-7 |
| Information  sources | 6 | Specify all databases, registers, websites, organisations, reference lists and other sources searched or consulted to identify studies. Specify the date  when each source was last searched or consulted. | 5-7 |
| Search strategy | 7 | Present the full search strategies for all databases, registers and websites, including any filters and limits used. | 5-7, Figure 1, Supplementary Material |
| Study selection  process | 8 | Specify the methods used to decide whether a study met the inclusion criteria of the review, including how many reviewers screened each record  and each report retrieved, whether they worked independently, and if applicable, details of automation tools used in the process. | 5-7 |
| Data collection process | 9 | Specify the methods used to collect data from study reports, including how many reviewers collected data from each report, whether they worked independently, any processes for obtaining or confirming data from study investigators, and if applicable, details of automat ion tools used in the  process. | 5-7 |
| Data Items | 10a | List and define all items for which data were sought from each study. | 5-7 |
|  | 10b | State the model performance measures that were sought (e.g., measures of calibration, discrimination, overall model fit, clinical utility). | 5-7, Table 1-3 |
|  | 10c | Describe how any desired but unreported data items (items 10a, 10b) were handled (e.g., contacted authors, calculated from other reported  information). | 5-7, Table 2 |
| Risk of bias and applicability  assessment | 11 | Specify the methods used to assess risk of bias in the included studies and their applicability to the review question. This should be done separately for each model development and validation. Include details of any tool(s) used, how many reviewers assessed each study and whether they worked  independently. | 6, 8, Supplementary Material |
| Synthesis methods | 12a | Describe any methods for synthesising estimates of performance measures for each model. If meta-analysis was carried out, describe the methods used, including any transformations of data prior to pooling, how any heterogeneity in model performance was quantified and handled, and  software package(s) used. | 5-7 |
|  | 12b | Describe any methods used to explore possible causes of heterogeneity in model performance (e.g., subgroup analysis, meta-regression), including  whether or not they were planned. | 5-7 |
|  | 12c | Describe any sensitivity analyses conducted to assess robustness of the synthesised results. | 6, 8, 9 |
| Certainty  assessment | 13 | Describe any methods used to assess certainty (or confidence) in the body of evidence for a prediction model. | 5, 6, 8, 9 |

**CHARMS 2014 checklist**

| **Domain** | **Key items** | **Reported on page #** |
| --- | --- | --- |
| **SOURCE OF DATA** | Source of data (e.g., cohort, case-control, randomized trial participants, or registry data) | 5-7, Table 1 |
| **PARTICIPANTS** | Participant eligibility and recruitment method (e.g., consecutive participants, location, number of centers, setting, inclusion and exclusion criteria) | 5, 6, Table 1 |
|  | Participant description | 5, 6, Table 1 |
|  | Details of treatments received, if relevant | Table 2 |
|  | Study dates | Table 1 |
| **OUTCOME(S) TO BE PREDICTED** | Definition and method for measurement of outcome | 5, 6 |
|  | Was the same outcome definition (and method for measurement) used in all patients? | 5,6, Table 3 |
|  | Type of outcome (e.g., single or combined endpoints) | 5, 6 |
|  | Was the outcome assessed without knowledge of the candidate predictors (i.e., blinded)? | Not applicable |
|  | Were candidate predictors part of the outcome (e.g., in panel or consensus diagnosis)? | 5-7, Table 2, Table 3 Supplementary material |
|  | Time of outcome occurrence or summary of duration of follow-up | 5, Table 3 |
| **CANDIDATE PREDICTORS**  **(OR INDEX TESTS)** | Number and type of predictors (e.g., demographics, patient history, physical examination, additional testing, disease characteristics) | Table 2 |
|  | Definition and method for measurement of candidate predictors | 5-7, Table 1-3 |
|  | Timing of predictor measurement (e.g., at patient presentation, at diagnosis, at treatment initiation) | 5-7, Table 2 |
|  | Were predictors assessed blinded for outcome, and for each other (if relevant)? | Not Applicable |
|  | Handling of predictors in the modelling (e.g., continuous, linear, non-linear transformations or categorised) | 5-7, Table 2, Table 3 Supplementary material |
| **SAMPLE SIZE** | Number of participants and number of outcomes/events | 5-7, Table 1-3 |
|  | Number of outcomes/events in relation to the number of candidate predictors (Events Per Variable) | 5-7, Table 1 |
| **MISSING DATA** | Number of participants with any missing value (include predictors and outcomes) | Not Reported |
|  | Number of participants with missing data for each predictor | Not Reported |
|  | Handling of missing data (e.g., complete-case analysis, imputation, or other methods) | Table 2 |
| **MODEL DEVELOPMENT** | Modelling method (e.g., logistic, survival, neural network, or machine learning techniques) | 5-7 |
|  | Modelling assumptions satisfied | 5-7 |
|  | Method for selection of predictors **for inclusion** in multivariable modelling (e.g., all candidate predictors, pre-selection based on unadjusted association with the outcome) | 5-7, Table 2 |
|  | Method for selection of predictors **during multivariable modelling** (e.g., full model approach, backward or forward selection) and criteria used (e.g., p-value, Akaike Information Criterion) | 5-7, Table 2 |
|  | Shrinkage of predictor weights or regression coefficients (e.g., no shrinkage, uniform shrinkage, penalized estimation) | Not Reported |
| **MODEL PERFORMANCE** | Calibration (calibration plot, calibration slope, Hosmer-Lemeshow test) and Discrimination (C-statistic, D-statistic, log-rank) measures with confidence intervals | 6-9, Figure 2, Figure 3 |
|  | Classification measures (e.g., sensitivity, specificity, predictive values, net reclassification improvement) and whether a-priori cut points were used | 6, Figure 2 and Figure 3 |
| **MODEL EVALUATION** | Method used for testing model performance: development dataset only (random split of data, resampling methods e.g. bootstrap or cross-validation, none) or separate external validation (e.g. temporal, geographical, different setting, different investigators) | 5-7, Table 2 |
|  | In case of poor validation, whether model was adjusted or updated (e.g., intercept recalibrated, predictor effects adjusted, or new predictors added) | 5-7, Table 2 |
| **RESULTS** | Final and other multivariable models (e.g., basic, extended, simplified) presented, including predictor weights or regression coefficients, intercept, baseline survival, model performance measures (with standard errors or confidence intervals) | 5-7, Table 1-3 |
|  | Any alternative presentation of the final prediction models, e.g., sum score, nomogram, score chart, predictions for specific risk subgroups with performance | Not Applicable |
|  | Comparison of the distribution of predictors (including missing data) for development and validation datasets | Table 2 |
| **INTERPRETATION AND DISCUSSION** | Interpretation of presented models (confirmatory, i.e., model useful for practice versus exploratory, i.e., more research needed) | 10-14 |
|  | Comparison with other studies, discussion of generalizability, strengths and limitations. | 10-14 |

**Appendix 3:**

**Supplementary Table 3 – Search String**

***Table 1 PICO Framework***

| **Population** | Patients with acute coronary syndrome |
| --- | --- |
| **Intervention** | Machine learning models |
| **Comparator** | Traditional methods |
| **Outcomes** | In-hospital mortality (comparative discrimination) |
| **Study design** | RCTs, observational trials |
| **Exclusion criteria** | Systematic reviews, case-series, letters, reviews, animal studies, non-English publications |

| **Number** | **Search Terms** | **Medline (PubMed)** | **Embase** | **Web of Science** | **Cochrane** |
| --- | --- | --- | --- | --- | --- |
| **1** | ((myocardial ischemia[MeSH Terms]) OR (myocardial ischemia[Title/Abstract]) OR (acute coronary syndrome[MeSH Terms]) OR (acute coronary syndrome*[Title/Abstract]) OR (ACS[Title/Abstract]) OR (ST-elevation*[Title/Abstract]) OR (non-ST elevation*[Title/Abstract]) OR (myocardial infarction[MeSH Terms]) OR (myocardial infarction[Title/Abstract]) OR (coronary artery disease[MeSH Terms]) OR (coronary artery disease[Title/Abstract]) OR (heart attack[Title/Abstract]) OR (percutaneous coronary intervention[MeSH Terms]) OR (percutaneous coronary intervention[Title/Abstract])) | **355,989** | **6,442** | **470,128** | **6,586** |
| **2** | ((machine learning*[Title/Abstract]) OR (machine learning[MeSH Terms]) OR (neural network*[Title/Abstract]) OR (random forest*[Title/Abstract]) OR (decision tree*[Title/Abstract]) OR (knowledge representation*[Title/Abstract]) OR (computer vision system*[Title/Abstract]) OR (computer reasoning*[Title/Abstract]) OR (natural language processing*[Title/Abstract]) OR (connectionist model*[Title/Abstract]) OR (expert system*)[Title/Abstract]) OR (artificial intelligence*[Title/Abstract]) OR (computat* intelligence*[Title/Abstract]) OR (computer* intelligence*[Title/Abstract]) OR (machine intelligence*[Title/Abstract]) OR (deep intelligence*[Title/Abstract]) OR (transfer intelligence*[Title/Abstract]) OR (hierarchical intelligence*[Title/Abstract]) OR (artificial learning*[Title/Abstract]) OR (computat* learning*[Title/Abstract]) OR (computer* learning*[Title/Abstract]) OR (deep learning*[Title/Abstract]) OR (transfer learning*[Title/Abstract]) OR (hierarchical learning*[Title/Abstract]) OR (artificial reasoning*[Title/Abstract]) OR (computat* reasoning*[Title/Abstract]) OR (computer* reasoning*[Title/Abstract]) OR (machine reasoning*[Title/Abstract]) OR (deep reasoning*[Title/Abstract]) OR (transfer reasoning*or hierarchical reasoning*[Title/Abstract]) OR (knowledge representation*[Title/Abstract]) OR (computer reasoning*[Title/Abstract]) OR (perceptron*[Title/Abstract]) OR (connectionist model*[Title/Abstract]) OR (expert system*[Title/Abstract]) OR (artificial intelligence*[Title/Abstract]) OR (computer* intelligence*[Title/Abstract]) OR (machine intelligence*[Title/Abstract]) OR (deep intelligence*[Title/Abstract]) OR (transfer intelligence*[Title/Abstract]) OR (hierarchical intelligence*[Title/Abstract]) OR (artificial learning*[Title/Abstract]) OR (computat* learning*[Title/Abstract]) OR (computer* learning*[Title/Abstract]) OR (machine learning*[Title/Abstract]) OR (deep learning*[Title/Abstract]) OR (transfer learning*[Title/Abstract]) OR (hierarchical learning*[Title/Abstract]) OR (artificial reasoning*[Title/Abstract]) OR (computat* reasoning*[Title/Abstract]) OR (computer* reasoning*[Title/Abstract]) OR (machine reasoning*[Title/Abstract]) OR (deep reasoning*[Title/Abstract]) OR (transfer reasoning*[Title/Abstract]) OR (hierarchical reasoning*[Title/Abstract])) | **610, 639** | **63893** | **541,629** | **6,5041** |
| **3** | 1 AND 2 | **4,189** | **2112** | **2,253** | **221** |

**Total = 6875**

After removal of duplicates (Endnote) = 5916

**Full text review = 39**

**Appendix 4: Justification of Exclusions at Full-Text Review**

**Wrong study design (n=11)**

1. Agasthi P, Ashraf H, Chao CJ, Wang PW, Allam M, Pujari SH, Fortuin FD, Eleid MF, Yang E, Beohar N, Mookadam F, Holmes DR, Arsanjani R. Machine Learning Helps Predict Short and Intermediate-term Risk for All Cause Mortality in Patients Undergoing Percutaneous Coronary Intervention. CIRCULATION. 2020;142. doi: doi:. PubMed PMID: rayyan-603673308.
2. Gallone G, Burrello J, Burrello A, Iannaccone M, De Luca L, Patti G, Cerrato E, Venuti G, De Filippo O, Mattesini A, Muscoli S, Trabattoni D, Giammaria M, Truffa A, Cortese B, Conrotto F, Mulatero P, Monticone S, Escaned J, Usmiani T, D'Ascenzo F, De Ferrari G, Breviario S. PREDICTION OF ALL-CAUSE MORTALITY FOLLOWING PERCUTANEOUS CORONARY INTERVENTION IN BIFURCATION LESIONS USING MACHINE LEARNING ALGORITHMS - THE RAIN-ML PREDICTION MODEL. EUROPEAN HEART JOURNAL SUPPLEMENTS. 2022;24. doi: doi:. PubMed PMID: rayyan-603676448.
3. Grant C, Raina A, Lennon R, Goodman SG, Gulati R, Lerman A, Rosenberg Y, So D, Farkouh ME, Athreya A, Pereira NL. Baseline Clinical and Genetic Data Based Machine Learning Predictions of 1-Year Ischemic Outcomes Following Percutaneous Coronary Intervention. CIRCULATION. 2021;144. doi: doi:. PubMed PMID: rayyan-603674857
4. Hamilton DE, Albright J, Seth M, Sukul D, Gurm HS. Merging Machine Learning and Patient Preference: A Contemporary, Comprehensive, Patient-Centered Tool for Risk Prediction Prior to Percutaneous Coronary Intervention. CIRCULATION. 2022;146. doi: doi:. PubMed PMID: rayyan-603676542.
5. Khan S, Zahid S, Zaid S, Kleiman NS. MACHINE LEARNING DERIVED RISK-PREDICTION MODEL FOR IN-HOSPITAL SURVIVAL IN PATIENTS UNDERGOING PERCUTANEOUS CORONARY INTERVENTION BEFORE ORGAN TRANSPLANT. JOURNAL OF THE AMERICAN COLLEGE OF CARDIOLOGY. 2023;81(8):827-. doi: doi:. PubMed PMID: rayyan-603679765.
6. Park JS. Machine Learning for Risk Prediction of Future Clinical Events in Patients With Acute Coronary Syndrome Undergone Percutaneous Coronary Intervention. CIRCULATION. 2019;140. doi: doi:. PubMed PMID: rayyan-603673036.
7. Sup J. Machine learning for risk pred future clinical events in patients with acute syndrome undergone percutaneous coronary intervention. Circulation. 2019;140. doi: doi:<https://doi.org/10.1161/circ.140.suppl1.9984>. PubMed PMID: rayyan-603673113.
8. Sup PJ. Machine Learning for Risk Prediction of Future Clinical Events in Patients With Acute Coronary Syndrome Who Have Undergone Percutaneous Coronary Intervention. JOURNAL OF THE AMERICAN COLLEGE OF CARDIOLOGY. 2019;74(13):B209-B. doi: doi:10.1016/j.jacc.2019.08.272. PubMed PMID: rayyan-603673115.
9. Sup PJ. TCT-210 Machine Learning for Risk Prediction of Future Clinical Events in Patients With Acute Coronary Syndrome Who Have Undergone Percutaneous Coronary Intervention. Journal of the American College of Cardiology. 2019;74(13):B209. doi: doi:<https://doi.org/10.1016/j.jacc.2019.08.272>. PubMed PMID: rayyan-603673114.
10. Yoshioka H, Hisaka A, Sato Y. Proposal of a new risk score for hospital death due to acute coronary syndrome developed by machine learning. Circulation research. 2020;127(12):e281. doi: doi:<https://doi.org/10.1161/RES.0000000000000450>. PubMed PMID: rayyan-603674346.
11. Zhou YJ, Zhu RY, Chen XJ, Xu XL, Wang QW, Jiang LJ, Zhu JH, Wu J, Yan H, Zhang L. MACHINE LEARNING-BASED CARDIOVASCULAR EVENT PREDICTION FOR PERCUTANEOUS CORONARY INTERVENTION. JOURNAL OF THE AMERICAN COLLEGE OF CARDIOLOGY. 2019;73(9):127-. doi: doi:10.1016/S0735-1097(19)30735-1. PubMed PMID: rayyan-603673293.

**No comparator (n=11)**

1. Burrello J, Gallone G, Burrello A, Jahier Pagliari D, Ploumen EH, Iannaccone M, De Luca L, Zocca P, Patti G, Cerrato E, Wojakowski W, Venuti G, De Filippo O, Mattesini A, Ryan N, Helft G, Muscoli S, Kan J, Sheiban I, Parma R, Trabattoni D, Giammaria M, Truffa A, Piroli F, Imori Y, Cortese B, Omedè P, Conrotto F, Chen SL, Escaned J, Buiten RA, Von Birgelen C, Mulatero P, De Ferrari GM, Monticone S, D'Ascenzo F. Prediction of All-Cause Mortality Following Percutaneous Coronary Intervention in Bifurcation Lesions Using Machine Learning Algorithms. J Pers Med. 2022;12(6). doi: doi:10.3390/jpm12060990. PubMed PMID: rayyan-603676163.
2. Cui S, Li L, Zhang Y, Lu J, Wang X, Song X, Liu J, Li K. Machine Learning Identifies Metabolic Signatures that Predict the Risk of Recurrent Angina in Remitted Patients after Percutaneous Coronary Intervention: A Multicenter Prospective Cohort Study. Adv Sci (Weinh). 2021;8(10):2003893. doi: doi:10.1002/advs.202003893. PubMed PMID: rayyan-603674709.
3. Deng L, Zhao X, Su X, Zhou M, Huang D, Zeng X. Machine learning to predict no reflow and in-hospital mortality in patients with ST-segment elevation myocardial infarction that underwent primary percutaneous coronary intervention. BMC Med Inform Decis Mak. 2022;22(1):109. doi: doi:10.1186/s12911-022-01853-2. PubMed PMID: rayyan-603676347.
4. Huang YC, Chen KY, Li SJ, Liu CK, Lin YC, Chen M. Implementing an Ensemble Learning Model with Feature Selection to Predict Mortality among Patients Who Underwent Three-Vessel Percutaneous Coronary Intervention. APPLIED SCIENCES-BASEL. 2022;12(16). doi: doi:10.3390/app12168135. PubMed PMID: rayyan-603676635
5. Kulkarni H, Amin AP. Artificial intelligence in percutaneous coronary intervention: improved risk prediction of PCI-related complications using an artificial neural network. BMJ INNOVATIONS. 2021;7(3):564-79. doi: doi:10.1136/bmjinnov-2020-000547. PubMed PMID: rayyan-603675063.
6. Liu S, Yang S, Xing A, Zheng L, Shen L, Tu B, Yao Y. Machine learning-based long-term outcome prediction in patients undergoing percutaneous coronary intervention. Cardiovasc Diagn Ther. 2021;11(3):736-43. doi: doi:10.21037/cdt-21-37. PubMed PMID: rayyan-603675199.
7. Matheny ME, Ohno-Machado L, Resnic FS. Discrimination and calibration of mortality risk prediction models in interventional cardiology. J Biomed Inform. 2005;38(5):367-75. doi: doi:10.1016/j.jbi.2005.02.007. PubMed PMID: rayyan-603669701.
8. Spertus JV, SL TN, Wolf R, Cioffi M, Lovett A, Rose S. Assessing Hospital Performance After Percutaneous Coronary Intervention Using Big Data. Circ Cardiovasc Qual Outcomes. 2016;9(6):659-69. doi: doi:10.1161/circoutcomes.116.002826. PubMed PMID: rayyan-603671572.
9. Subhan S, Malik J, Haq AU, Qadeer MS, Zaidi SMJ, Orooj F, Zaman H, Mehmoodi A, Majeedi U. Role of Artificial Intelligence and Machine Learning in Interventional Cardiology. Curr Probl Cardiol. 2023;48(7):101698. doi: doi:10.1016/j.cpcardiol.2023.101698. PubMed PMID: rayyan-603680431.
10. Wang J, Wang S, Zhu MX, Yang T, Yin Q, Hou Y. Risk Prediction of Major Adverse Cardiovascular Events Occurrence Within 6 Months After Coronary Revascularization: Machine Learning Study. JMIR Med Inform. 2022;10(4):e33395. doi: doi:10.2196/33395. PubMed PMID: rayyan-603678389.
11. Wang Y, Zhu K, Li Y, Lv Q, Fu G, Zhang W. A machine learning-based approach for the prediction of periprocedural myocardial infarction by using routine data. Cardiovasc Diagn Ther. 2020;10(5):1313-24. doi: doi:10.21037/cdt-20-551. PubMed PMID: rayyan-603674263.

**Wrong population (n=3)**

1. Farhadian M, Dehdar Karsidani S, Mozayanimonfared A, Mahjub H. Risk factors associated with major adverse cardiac and cerebrovascular events following percutaneous coronary intervention: a 10-year follow-up comparing random survival forest and Cox proportional-hazards model. BMC Cardiovasc Disord. 2021;21(1):38. doi: doi:10.1186/s12872-020-01834-1. PubMed PMID: rayyan-603674792.
2. Ninomiya K, Kageyama S, Garg S, Masuda S, Kotoku N, Revaiah PC, O'Leary N, Onuma Y, Serruys PW. Can machine learning unravel unsuspected, clinically important factors predictive of long-term mortality in complex coronary artery disease? A call for 'big data'. Eur Heart J Digit Health. 2023;4(3):275-8. doi: doi:10.1093/ehjdh/ztad014. PubMed PMID: rayyan-603680114.
3. Sampedro-Gómez J, Dorado-Díaz PI, Vicente-Palacios V, Sánchez-Puente A, Jiménez-Navarro M, San Roman JA, Galindo-Villardón P, Sanchez PL, Fernández-Avilés F. Machine Learning to Predict Stent Restenosis Based on Daily Demographic, Clinical, and Angiographic Characteristics. Can J Cardiol. 2020;36(10):1624-32. doi: doi:10.1016/j.cjca.2020.01.027. PubMed PMID: rayyan-603674061.

**Full text unavailable (n=1)**

1. Lee G, Gurm HS, Syed Z. Predicting complications of percutaneous coronary intervention using a novel support vector method. J Am Med Inform Assoc. 2013;20(4):778-86. doi: doi:10.1136/amiajnl-2012-001588. PubMed PMID: rayyan-603670746.

**Appendix 5:**

**Supplementary Table 4: Risk of bias assessment for included studies using the PROBAST risk of bias tool**

| **Study** | **Model** |  | **ROB** | |  | **Overall** |
| --- | --- | --- | --- | --- | --- | --- |
|  |  | **Participants** | **Predictors** | **Outcome** | **Analysis** | **ROB** |
| Calburean 2019 | CatBoost | 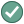 | 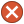 | 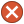 | 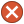 | 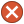 |
| Chao 2023 | RF | 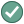 | 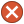 | 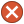 | 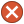 | 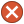 |
| Doll 2021 | CART PCI Mortality Model | 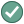 | 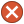 | 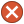 | 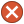 | 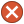 |
| Li 2023 | DNN | 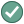 | 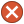 | 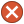 | 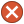 | 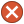 |
| Mortazavi 2019 | XGBoost | 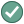 | 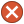 | 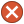 | 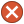 | 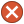 |
| Niimi 2022 | XGBoost | 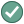 | 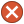 | 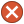 | 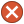 | 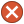 |
| Rayfield 2020 | AL-BR | 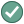 | 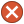 | 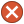 | 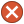 | 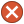 |
| Resnic 2001 | Simplified Prognostic Risk Scoring Systems | 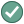 | 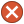 | 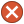 | 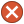 | 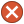 |
| Sampedro 2020 | LR | 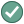 | 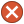 | 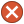 | 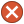 | 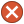 |
| Shi 2023 | GRACE | 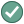 | 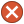 | 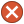 | 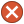 | 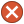 |
| Xiao 2022 | RF | 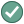 | 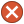 | 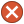 | 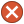 | 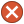 |
| Zack 2019 | LR | 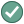 | 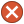 | 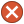 | 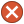 | 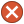 |
| Zhao 2023 | XG Boost | 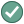 | 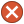 | 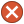 | 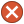 | 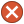 |

**PROBAST Risk of Bias assessment for machine learning models in the included studies.**
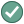
 - Low risk of bias or good applicability,
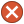
 - High risk of bias or low applicability,
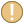
 - Unclear risk of bias or applicability.
